# Carbon nanotubes as the supporting film for cryo-EM specimen preparation

**DOI:** 10.1038/s41467-026-77953-8

**Published:** 2026-09-21

**Authors:** Jiling Song, Zhengni Wang, Duanliang Zhou, Zi Yuan, Xia Jia, Xiaopeng Li, Chenhui Zhang, Yongbo Liu, Yu Rao, Dongju Wang, Kaili Jiang, Hong-Wei Wang, Nan Liu

**Affiliations:** 1 School of Life Sciences, Tsinghua University, Beijing, China; 2 Ministry of Education Key Laboratory of Protein Sciences, Tsinghua University, Beijing, China; 3 Beijing Frontier Research Center for Biological Structures, Tsinghua University, Beijing, China; 4 State Key Laboratory of Low Dimensional Quantum Physics, Department of Physics, Tsinghua University, Beijing, China; 5 Frontier Science Center for Quantum Information, Department of Physics, Tsinghua University, Beijing, China; 6 Tsinghua-Peking Joint Center for Life Sciences, Tsinghua University, Beijing, China; 7 School of Biological Sciences, The University of Hong Kong, Hong Kong, China; 8 School of Pharmaceutical Sciences, Tsinghua University, Beijing, China

**Keywords:** Electron microscopy, Structure determination, Structural biology, Biophysics, Biochemistry

## Abstract

Specimen preparation is a critical and rate-limiting step in cryo-EM structural analysis, primarily due to challenges posed by the air-water interface. In this study, we introduce carbon nanotubes (CNTs) as a one-dimensional fibrous support material to address this issue. CNTs interact with macromolecules, effectively protecting them from air-water interfaces, and macromolecules arrange themselves around CNTs in various orientations. Furthermore, CNTs biofunctionalized with affinity tags exhibit strong specificity in binding target macromolecules, facilitating direct purification of targets from cell lysates and on-grid assembly of complexes for subsequent cryo-EM analysis. We also demonstrate that CNT grids are compatible with cell culturing, allowing for seamless integration into the cryo-EM characterization workflow for cellular structures. We anticipate that these properties will make the CNT network a promising support for cryo-EM.

## Abstract (web summary)

Cryo-EM specimen preparation is often hindered by air–water interface. Here, authors develop carbon nanotube supports that protect macromolecules and enable affinity purification, providing a versatile platform for structural studies.

## Introduction

Cryo-EM specimen preparation has gained significant attention due to its crucial role in structural analysis^1^. In the conventional procedure, the sample solution on the EM grid is thinned by blotting with filter papers and then rapidly frozen at liquid nitrogen temperature. A major challenge in this process is the interference from the air-water interface. Numerous cryo-EM studies have shown that macromolecules in the specimen are primarily distributed at the air-water interfaces, which can lead to sample denaturation and preferential orientation, ultimately compromising the final 3D reconstruction^2,3^. It is now recognized that the strong interaction between macromolecules and air-water interfaces occurs because the time it takes for macromolecules to reach the air-water interfaces of the thin liquid film—less than a millisecond—is much shorter than the time between the formation of the thin liquid film on the EM grid and its vitrification at liquid nitrogen temperature, which typically takes several seconds^4,5^.

Recent advancements in cryo-EM methodology have introduced various strategies to optimize specimen preparation. Supporting films, such as graphene and its derivatives^6–9^, provide a robust interaction interface with minimal background noise, effectively improving the behavior of target macromolecules in vitreous ice. Protein- or detergent-formed 2D supporting films^10–14^ have also been proven effective in mitigating issues related to the air-water interface and preferential orientation. Supporting films functionalized with affinity ligands can anchor target macromolecules, keeping them away from the air-water interface and purifying them from impure systems or even directly from cell lysates. This integration of sample purification with specimen preparation, referred to as “purification/assembly-on-grid”^15–17^, also addresses the substantial sample loss typically observed during the filter paper blotting process. However, non-specific binding remains a challenging issue to resolve.

Inspired by the idea of minimizing the time macromolecules spend reaching the air-water interface, new specimen preparation devices, such as Vitrojet^18^ and Chameleon^19^, have been developed, with some already available commercially. These devices can rapidly vitrify sample solutions in just tens of milliseconds^20,21^, and require only a sub-nanoliter quantity of solution, which is deposited onto EM grids and spread into a thin liquid film. A recent approach involves spraying a small sample droplet at high speed onto EM grids that are pre-cooled to liquid ethane temperature, allowing the sample to be thinned and vitrified within microseconds^22^. This method eliminates the exposure of protein particles to the air–water interface. A key aspect of using these devices for cryo-EM specimen preparation is ensuring that the solution spreads evenly and forms a thin liquid film on the grids^23^, which has not always been achieved reliably.

Carbon nanotubes (CNTs) are a carbon family allotrope with a one-dimensional spiral structure formed by single or multiple layers of graphene rolled together^24^, exhibiting high mechanical strength, excellent electrical conductivity, and chemical properties^25^.

**Fig. 1:**
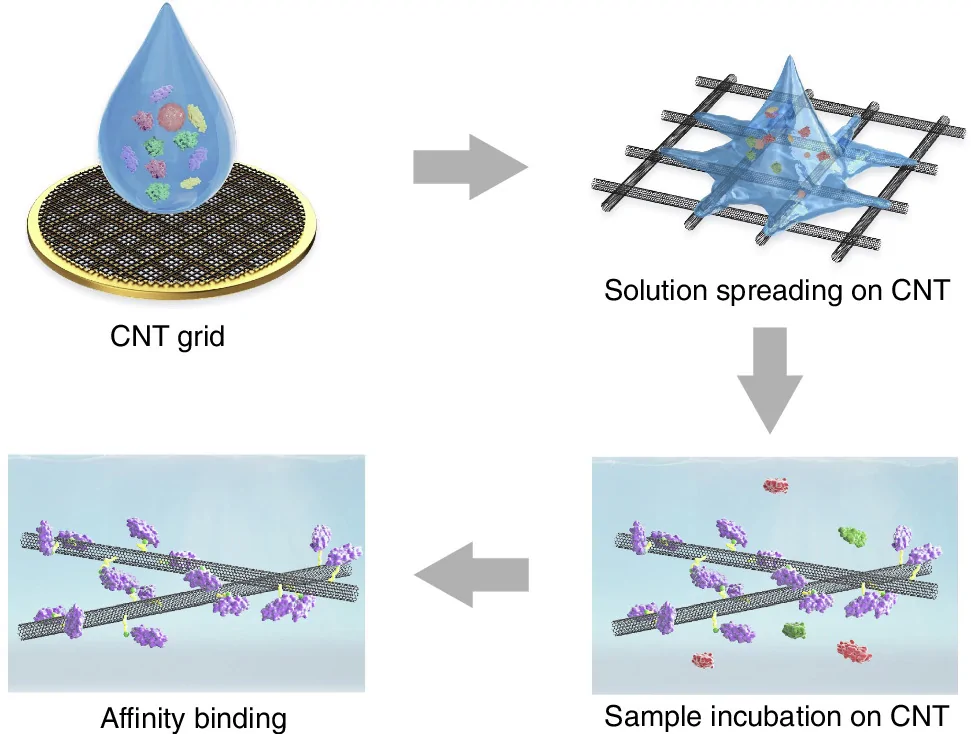
Schematic of using the CNT grid for cryo-EM structural analysis. Carbon nanotubes (CNTs) generate capillary action that helps the spreading of the sample solution. Affinity CNTs can anchor target protein particles in various orientations, enabling on-grid purification or assembly of targets for cryo-EM structural determination.

In this study, we fabricate CNT films spun from super-aligned carbon nanotube (SACNT) arrays^26,27^. These CNT films, with a typical thickness of tens of nanometers, are utilized as the supporting film for cryo-EM specimen preparation (Fig. 1). When treated to become hydrophilic, CNTs facilitate capillary action, aiding in the spreading of the sample solution on EM grids. As one-dimensional support, CNT films allow macromolecules to orient themselves around CNTs in all directions, thereby minimizing interference from the air-water interface and enriching the macromolecule orientations. Furthermore, we have characterized different strategies for modifying CNTs to manipulate interactions with biomolecules, leading to an effective platform utilized for on-grid purification and/or assembly of target protein complexes directly from cell lysates. Notably, CNTs also demonstrate good biocompatibility for cell culture, allowing them to serve as a support for the cryo-EM characterization of in situ cellular structures.

## Results

### The fabrication and use of CNT grids

**Fig. 2:**
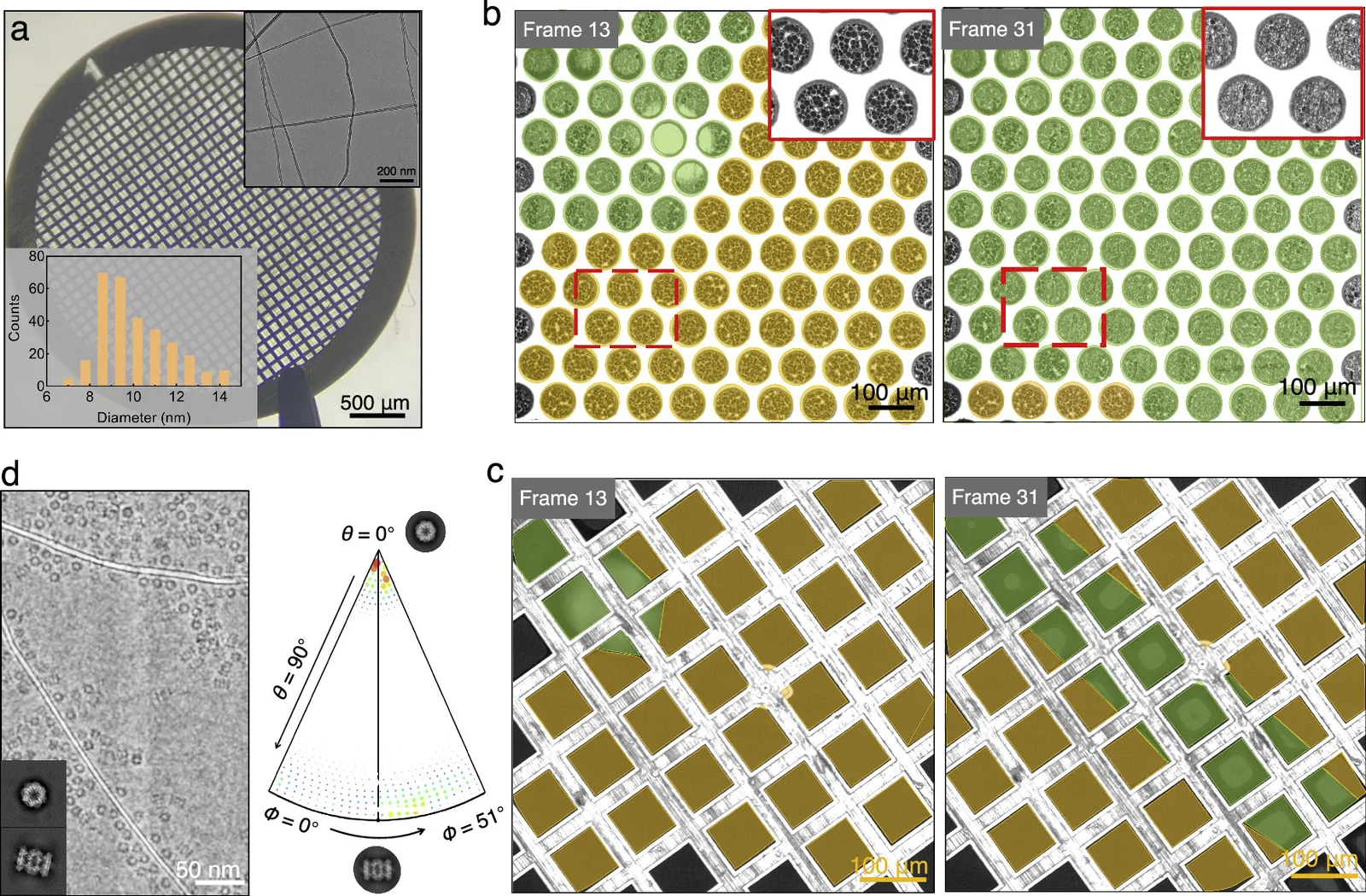
CNT grid for preparing cryo-EM specimens. **a** Optical microscopy image of a 300 mesh TEM grid coated with CNTs. The insets show the TEM characterization (upper right) and the statistics of CNT diameter on EM grids (bottom left). For statistics of the diameter of carbon nanotubes, 300 CNTs were measured. **b**,** c** Two sequential frames recorded 1.7 s apart on the VitroJet illustrate the process of fluid movement across a CNT grid (**b**) or a conventional holey carbon-coated EM grid (**c**). Insets in (**b**) provide enlarged views of the same area, showing a non‑wetted state (left) and a fully wetted state (right). For clarity, fluid‑filled regions are marked in green, while non-wetted regions are in orange; raw images are available in Supplementary Fig. 5. Three grids were tested for each group independently with similar results. **d** Representative cryo-EM micrograph of the 20S proteasome on a CNT grid (left), alongside the corresponding 2D class averages and the particle orientation distribution derived from this dataset (right). In the particle orientation distribution plot, the Euler angles *θ* and *Φ* define the orientation of each particle relative to the reference model during 3D alignment; larger spot sizes colored in red indicate more particles assigned to that view. Source data are provided as a Source Data file.

The CNT arrays were synthesized on a silicon wafer using a low-pressure chemical vapor deposition method^27^, spun to form crossed CNT films, and then transferred onto EM grids (Fig. 2a and Supplementary Fig. 1, see Methods for details)^28,29^. The diameter of the CNTs on the EM grid was ~10 nm. To ensure the density of CNTs is compatible with subsequent cryo-EM sample preparation and imaging, optimal transmission assessment was performed. In our practice, CNTs with a transmission of ~60% at a wavelength of 550 nm were recommended, and the pre-strained treatment did not weaken the mechanical strength of CNTs (Supplementary Fig. 2, 3). Initially, the CNT grids exhibited significant hydrophobicity, with a water contact angle exceeding 123° (Supplementary Fig. 4). To render the CNT grids hydrophilic, we applied a low-energy oxygen plasma treatment. This method proved highly effective: after a 20 s treatment, the water contact angle decreased to 41.8° (Supplementary Fig. 4). Further increasing the treatment duration enhanced the hydrophilicity, achieving a water contact angle of 13.2° after a 60-second treatment, making CNT grids suitable for the loading and spreading of sample solution.

The spreading of the sample solution across the EM grid is crucial for achieving optimal ice thickness and sample distribution, particularly in the blotting-free specimen preparation methods^18,19^. To characterize the solution spreading on the CNT grids, we used the VitroJet machine^18^ to apply a tiny droplet onto a plasma-treated CNT grid, in which a pin carrying a sub-nanoliter volume of solution wrote onto the grid, creating a liquid footprint. We found that the solution spread rapidly beyond the pin writing trace upon contacting the CNTs, which is attributed to the significant capillary action generated by the hydrophilic CNTs^30^ (Fig. 2b, Supplementary Video 1, and Supplementary Fig. 5). In contrast, on the conventional plasma-treated EM grid that is coated with holey carbon film, we did not observe noticeable solution spreading, with the liquid remaining confined to the area where the pin made contact (Fig. 2c, Supplementary Video 2 and Supplementary Fig. 5).

Using CNT grids, we prepared cryo-EM specimens of the 20S proteasome (Supplementary Fig. 6). The particles were predominantly localized near the CNTs (Fig. 2d), rather than being uniformly distributed as on conventional grids (Supplementary Fig. 7a). Subsequent single-particle analysis revealed a diverse particle angular distribution on the CNT grids, including both top (round) and side (rectangular) views. This contrasts with the predominance of side views observed on conventional grids (Supplementary Fig. 7b). The tomogram reconstruction of the cryo-specimen revealed that the CNTs were embedded within the vitreous ice, remaining distant from both air-water interfaces (Supplementary Fig. 8), and most 20S proteasome particles were situated close to the CNTs. This data indicates that CNTs help position proteins in various orientations within the vitreous ice, unlike samples prepared on conventional EM grids.

### Affinity functionalization of CNT grids

**Fig. 3:**
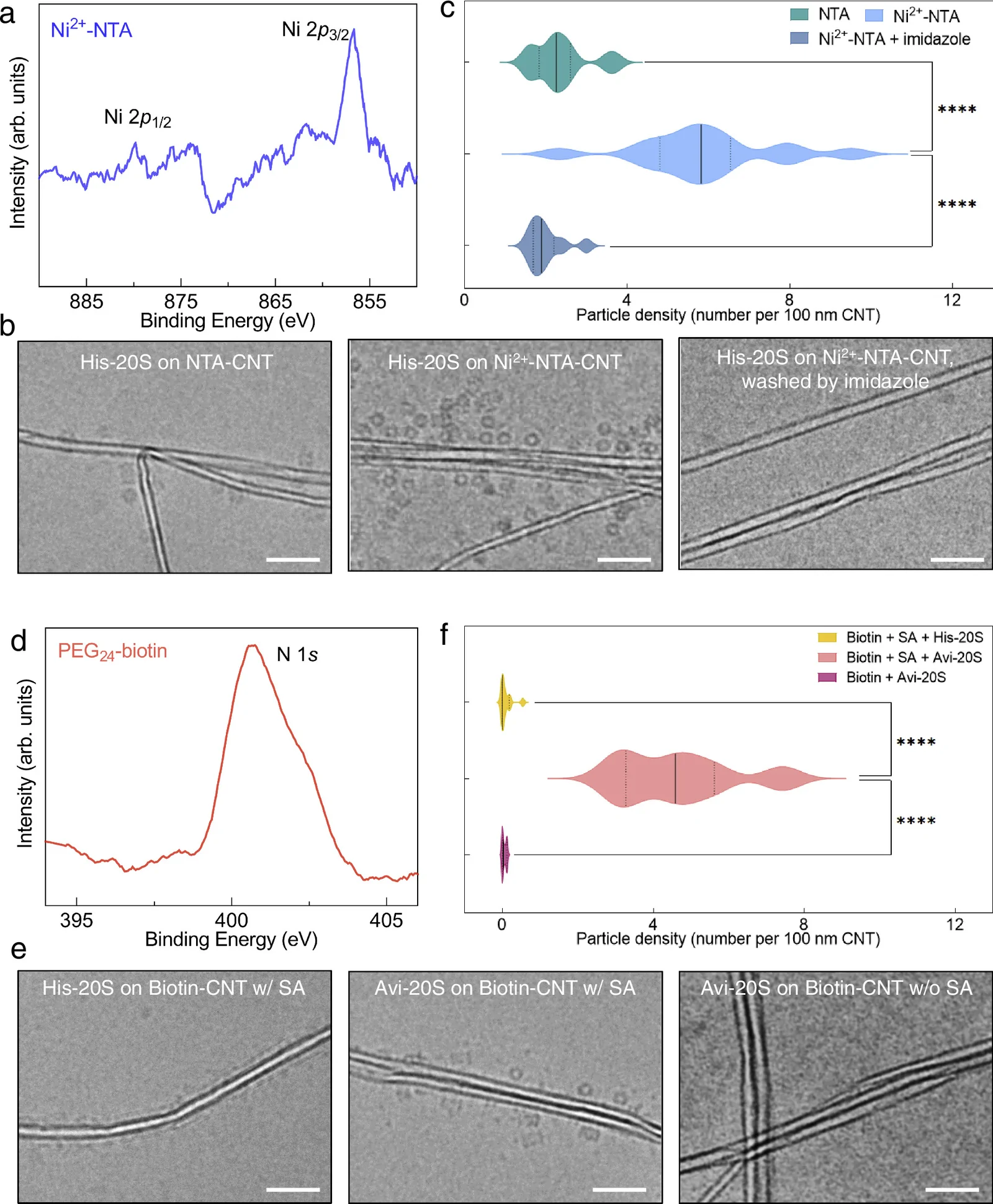
Functionalized CNT grids enable specific binding with target proteins. **a** X-ray photoelectron spectra of Ni^2+^-NTA-modified CNTs; the corresponding survey spectrum is shown in Supplementary Fig. 9a. **b** Representative cryo-EM images of CNT grids after incubation with His₆‑tagged 20S proteasome, followed by washing steps. From left to right: NTA‑modified CNTs washed with buffer; Ni²⁺‑NTA‑modified CNTs washed with buffer; and Ni²⁺‑NTA‑modified CNTs washed with imidazole. Scale bars, 50 nm. **c** Statistical results of 20S proteasome particle density along the CNTs in (**b**), defined as the number of particles per 100 nm CNT. The solid lines in the violin plot indicate the median, and the dotted lines represent the first quartile (lower quartile) and the third quartile (upper quartile). Two-sided *t* test was used; ****p < 0.0001. **d** X-ray photoelectron spectra of PEG_24_-biotin-modified CNTs; the corresponding survey spectrum is shown in Supplementary Fig. 9b. **e** Representative cryo‑EM images of PEG₂₄‑biotin‑modified CNTs after: (left) sequential incubation with streptavidin and His₆‑tagged 20S proteasome; (middle) sequential incubation with streptavidin and Avi‑tagged 20S proteasome; (right) direct incubation with Avi‑tagged 20S proteasome. Scale bars, 50 nm. **f** Statistical results of 20S proteasome particle density along the CNTs in (**e**) defined as the number of particles per 100 nm CNT. The solid lines in the violin plot indicate the median, and the dotted lines represent the first quartile (lower quartile) and the third quartile (upper quartile). Two-sided *t* test was used; ****p < 0.0001. Source data are provided as a Source Data file.

To endow the CNTs with specific binding capacity, we next sought to functionalize the CNTs with various affinity ligands, such as Ni²⁺-NTA or PEG_24_-biotin, using our previously established protocols^31,32^. The functionalization results were first characterized by X-ray photoelectron spectroscopy (XPS) analysis. It revealed Ni 2*p*_3/2_ and Ni 2*p*_1/2_ signals from Ni²⁺-NTA-modified CNTs and N 1 *s* signals from PEG_24_-biotin-modified CNTs (Fig. 3a, d and Supplementary Fig. 9). Raman spectrum analysis (Supplementary Fig. 10) also showed an increase in the *D*/*G* intensity ratio for CNTs modified with Ni²⁺-NTA or PEG_24_-biotin, compared to the non-modified ones. Specifically, the *D*/*G* intensity ratio was 0.83 for Ni²⁺-NTA-modified CNTs, 0.91 for PEG_24_-biotin-modified CNTs, and 0.53 for non-modified CNTs. These results collectively indicated the successful functionalization of the CNT grids with these ligands.

We then applied His_6_-tagged 20S proteasome particles to Ni²⁺-NTA-modified CNT grids and performed cryo-EM analysis, using NTA-modified CNT grids as the control. Briefly, we incubated the CNT grids with 20S proteasome, washed them with buffer solution (50 mM Tris-HCl, 500 mM NaCl, pH 7.5) to remove non-specific binding particles, and then prepared the cryo-EM specimen. In the control group (without Ni²⁺), only a few protein particles adhered to CNTs (Fig. 3b, left). In contrast, numerous 20S proteasome particles were found distributed along the Ni²⁺-NTA-modified CNTs in various orientations (Fig. 3b, middle and Supplementary Fig. 11), and after being washed with 500 mM imidazole solution, the number of particles along the CNTs notably decreased (Fig. 3b, right). For statistical analysis of the specific binding affinity, we counted the particle numbers per 100 nm of CNT length (denoted as particle density) across 200 nanotubes for each group (Fig. 3c). On average, 5.9 particles per 100 nm on the Ni²⁺-NTA-modified CNTs were observed, and after being washed with 500 mM imidazole, 2.0 particles per 100 nm remained, which was comparable to the 2.4 particles per 100 nm on the NTA-modified CNTs group. These grids were further collected and subjected to SDS-PAGE analysis with silver staining, yielding consistent results (Supplementary Fig. 11a).

To assess the specific binding affinity of the PEG_24_-biotin-modified CNT grids, we first immobilized streptavidin on the grids as the mediator, a process referred to as streptavidin activation of the PEG_24_-biotin-modified CNTs. We then incubated the streptavidin-activated CNT grids with Avi-tagged or His_6_-tagged 20S proteasome, with the latter as a control group, followed by washing with buffer solution (25 mM Tris-HCl, 500 mM NaCl, pH 8.0) (Fig. 3e). No His_6_-tagged 20S proteasome particles were observed on the streptavidin-activated CNTs; instead, small dot-like densities were seen along the CNTs (Fig. 3e, left), likely corresponding to the streptavidin particles. In contrast, when incubated with Avi-tagged 20S proteasome particles, numerous 20S proteasome particles with various orientations were observed to adhere to the streptavidin-activated CNTs (Fig. 3e, middle). To confirm that the binding of Avi-tagged 20S proteasome was mediated by streptavidin, we also directly incubated PEG_24_-biotin-modified CNTs with Avi-tagged 20S proteasome without prior streptavidin activation. In this group, the dot-like densities along the CNTs observed in the streptavidin-activated groups were absent, and almost no 20S proteasome particles were found along the CNTs (Fig. 3e, right). This result confirmed that the dot-like densities along the streptavidin-activated CNTs (Fig. 3e, left) represented streptavidin particles and that the Avi-tagged 20S proteasome particles specifically bound to the streptavidin-activated CNTs through the mediation of streptavidin. Statistical analysis (Fig. 3f) revealed that His_6_-tagged 20S proteasome particles had a very low binding affinity to streptavidin-activated CNTs, averaging 0.1 particles per 100 nm of CNTs. Similarly, Avi-tagged 20S proteasome exhibited low binding affinity on PEG_24_-biotin-modified CNTs without streptavidin activation, with an average of 0.05 particles per 100 nm of CNTs. However, when activated by streptavidin, Avi-tagged 20S proteasome showed a significantly higher binding affinity to CNTs, averaging 4.6 particles per 100 nm of CNTs. These results collectively demonstrate that the CNT grid can be affinity-functionalized and exhibit high binding specificity to targets.

### Controllable particle-CNT interaction via functionalization setup

**Fig. 4:**
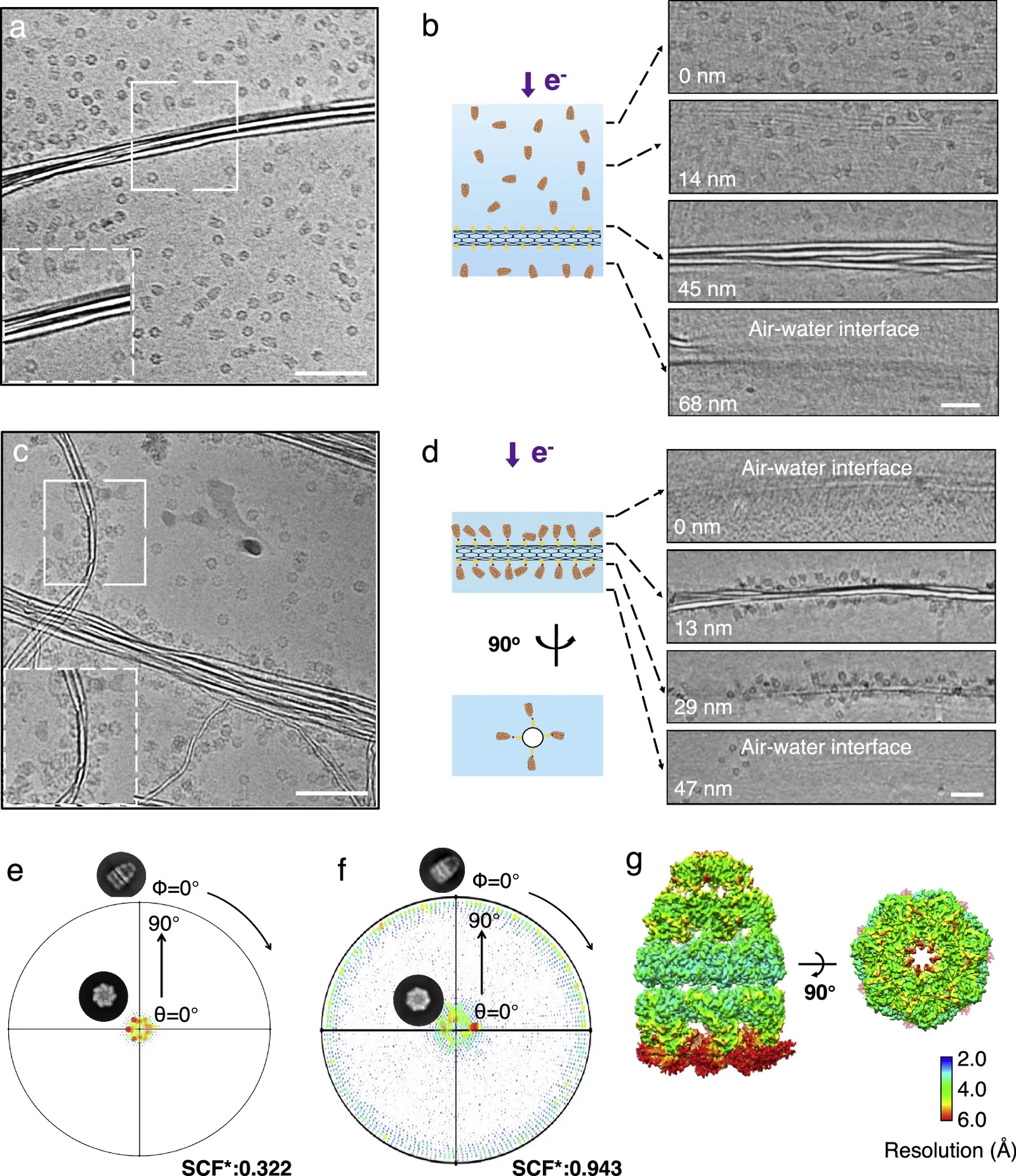
Interactions between particles and affinity functionalized CNTs. **a** Representative cryo-EM image of PEG_24_-biotin-modified CNTs incubated with Avi-tagged GroES/GroEL complex. Scale bar, 100 nm. More than 20 micrographs were captured independently with similar results. **b** Four slices from the corresponding cryo-ET reconstruction show that particles are evenly distributed in the open regions, rather than along the CNTs. Scale bar, 50 nm. More than 10 tilt-series were collected independently with similar results. **c** Representative cryo-EM image of streptavidin-activated CNTs, incubated sequentially with Avi-tagged GroES, and then GroEL. Scale bar, 100 nm. More than 20 micrographs were captured independently with similar results. **d** Selected slices from the cryo-ET reconstruction, spanning 0 nm to 47 nm from the upper boundary, show that particles are anchored along the CNTs. Scale bar, 50 nm. More than 10 tilt-series were collected independently with similar results. **e**,** f** Euler‑angle distributions of particles in the open regions (**e**) and along the streptavidin‑activated CNTs (**f**); larger, redder spots indicate a higher particle count in a given orientation. The Sampling Compensation Factors (SCF) are also indicated. **g** Cryo-EM density map of the GroES/GroEL complex along the streptavidin-activated CNTs, colored according to the local resolution distribution.

The aforementioned results demonstrate that CNTs functionalized with PEG_24_-biotin exhibited minimal non-specific particle binding compared to both Ni²⁺-NTA-modified and non-modified CNTs (Fig. 3), suggesting that PEG_24_-biotin modification effectively reduces the non-specific interactions. To further verify this, we applied the GroES/GroEL complex—with the GroES subunit carrying an Avi tag—onto the PEG_24_-biotin-modified CNT grid without streptavidin activation treatment, followed by cryo-EM imaging (Fig. 4a and Supplementary Fig. 12). We found that all the GroES/GroEL particles were excluded from the CNT-proximal regions and were primarily distributed in the open regions (Fig. 4a and Supplementary Fig. 12a). After washing with buffer solution, almost no particles remained (Supplementary Fig. 12b). The cryo-ET reconstruction further confirmed that the particles were evenly distributed in the open regions away from the CNTs (Fig. 4b). These results indicate that PEG_24_-biotin modification effectively eliminates the non-specific binding of proteins.

We next activated the PEG_24_-biotin-modified CNT grid with streptavidin and then incubated the grid with Avi-tagged GroES, followed by GroEL subunits. We observed a high density of assembled GroES/GroEL complexes along the CNTs (Fig. 4c). The cryo-ET analysis clearly showed that the particles were distributed along the CNTs within the vitreous ice (Fig. 4d). We further performed the single-particle reconstruction using the GroES/GroEL particles in the open regions (Fig. 4e) or along the streptavidin-activated CNTs (Fig. 4f). We found that GroES/GroEL predominantly adopted a top view (round shape) in the open regions; further cryo-ET analysis was consistent with this (Supplementary Fig. 12c). The Sampling Compensation Factor (SCF), which measures the effect of orientation distribution on the spectral signal-to-noise ratio and reflects orientational efficiency^33,34^, was calculated to be only 0.322 for GroES/GroEL particles in the open regions (Fig. 4e). In contrast, structural reconstruction of the GroES/GroEL complex using particles along the streptavidin-activated CNT grid not only validated on-grid assembly of the GroES/GroEL complex, but also showed a much more diverse orientational distribution than that in the open regions, with a much higher SCF value of 0.943 (Fig. 4f).

Additionally, the local resolution distribution of the GroES/GroEL reconstruction derived from streptavidin-activated CNTs indicated that one end of the complex—the end opposite to GroES—exhibited significantly lower resolution than the overall map (Fig. 4g). This contrasts with various existing cryo-EM reconstructions of the GroES/GroEL complex, which typically display lower resolution at both ends relative to the overall structure^35^. One explanation is that in our setup, the Avi-tagged GroES subunit is anchored inside the vitreous ice via streptavidin on the CNTs. This leaves its opposite end more exposed to the air-water interface, potentially leading to structural distortion. We also observed some protein aggregation along the carbon nanotubes (Fig. 4c), which may result from suboptimal interactions at the CNT-water interface. Consequently, these results provide direct evidence that the air-water interface can compromise cryo-EM structural determination and that functionalized CNTs could mitigate this effect.

### Affinity CNTs enable an effective on-grid purification

**Fig. 5:**
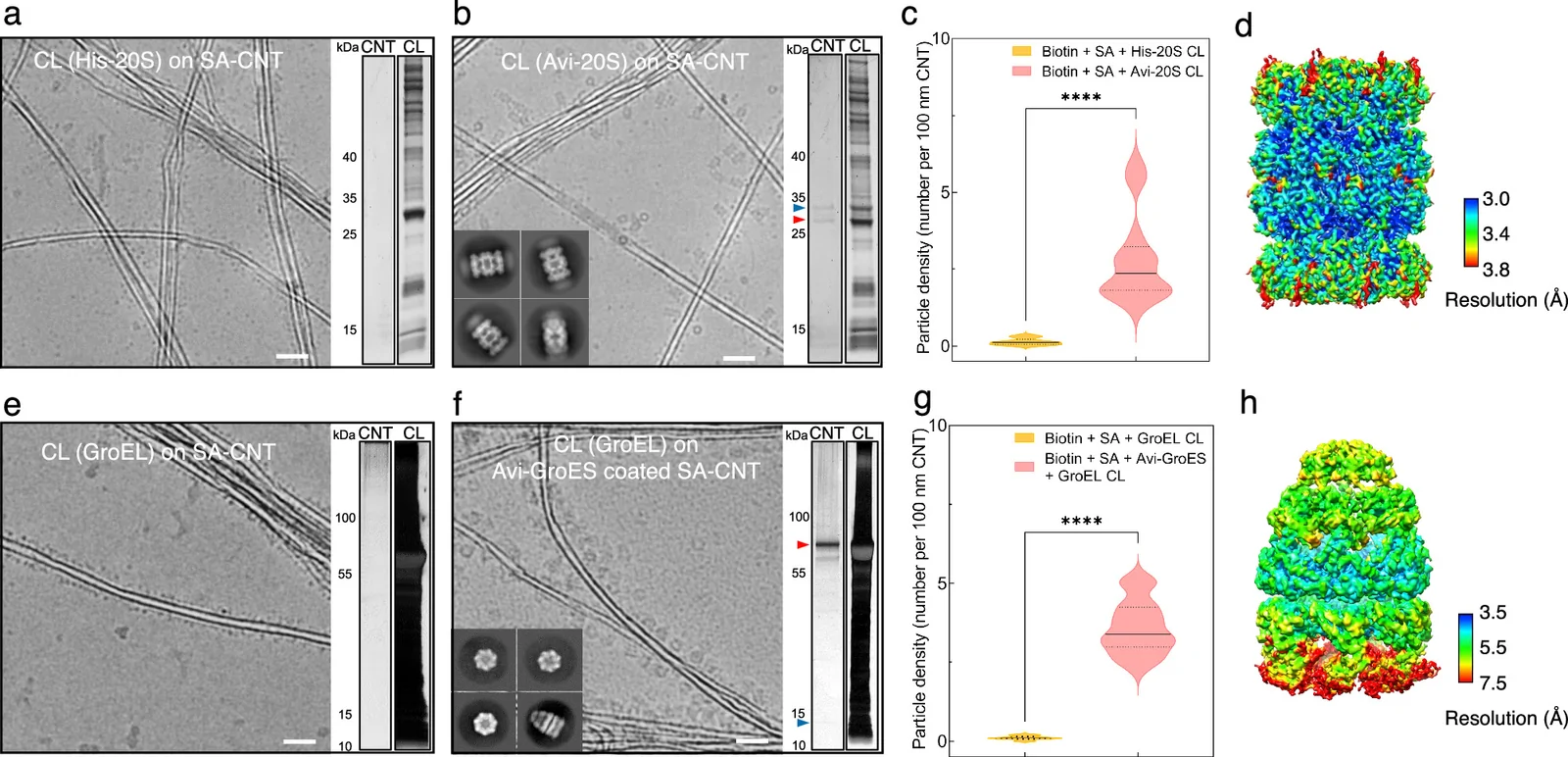
Affinity functionalized CNTs enable on-grid purification and complex assembly. **a**,** b** Representative cryo-EM images of streptavidin-activated CNTs (SA-CNT) incubated with cell lysates (CL) containing His_6_-tagged (**a**) or Avi-tagged (**b**) 20S proteasome. Adjacent silver‑stained SDS‑PAGE gels show the input lysates and corresponding CNT captures. The α and β subunits of the 20S proteasome are marked with blue and red arrows, respectively. Scale bar, 100 nm. **c** Statistical results of 20S proteasome particle density along the CNTs in (**a**, **b**), defined as the number of particles per 100 nm CNT. The solid lines in the violin plot indicate the median, and the dotted lines represent the first quartile (lower quartile) and the third quartile (upper quartile). Two-sided *t* test was used; ****p < 0.0001. **d** Cryo-EM density map of the 20S proteasome, reconstructed using particles in (**b**), with colors indicating the local resolution distribution. **e**,** f** Representative cryo-EM images of streptavidin-activated CNTs incubated with cell lysates containing GroEL (**e**) or Avi-tagged GroES followed by cell lysates containing GroEL (**f**). GroES was indicated by a blue arrow in the SDS-PAGE gels, while GroEL was marked with a red arrow on the silver-stained gels. Scale bar, 100 nm. **g** Statistical results of GroES/GroEL complex particle density along the CNTs in (**e**,** f**) defined as the number of particles per 100 nm of the CNT. The solid lines in the violin plot indicate the median, and the dotted lines represent the first quartile (lower quartile) and the third quartile (upper quartile). Two-sided *t* test was used; ****p < 0.0001. **h** Cryo-EM density map of the GroES/GroEL complex, reconstructed using particles in (**f**), with colors indicating the local resolution distribution. Source data are provided as a Source Data file.

Due to their high binding specificity, we hypothesized that affinity-functionalized CNTs could be used to directly purify target proteins from cell lysates, namely on-grid purification (Supplementary Fig. 13). To test this, we first performed a control experiment in which the streptavidin-activated CNT grid was incubated with the *E. coli* cell lysate containing overexpressed His_6_-tagged, instead of Avi-tagged, 20S proteasome, followed by cryo-EM characterization (see Methods for details). The results showed minimal capture of proteasome particles, with only small dots visible along the CNTs (Fig. 5a). We further used silver staining SDS-PAGE to quantify the capture efficiency of the CNT grid. Consistent with the cryo-EM analysis, no bands corresponding to 20S proteasome subunits were detected in the gels (Fig. 5a).

When we incubated cell lysates containing overexpressed Avi-tagged 20S proteasome with streptavidin-activated CNTs, distinct proteasome particles were visible in the raw micrographs (Fig. 5b). Two-dimensional classification of these particles revealed additional density at both ends of the proteasome, which corresponded to streptavidin, confirming that the proteasomes were bound to the streptavidin-activated CNTs. Silver staining SDS-PAGE analysis further validated the presence of 20S proteasome subunits, supporting the capability of streptavidin-activated CNTs to isolate Avi-tagged proteasome from impure lysates (Fig. 5b). Further statistical analysis showed that streptavidin-activated CNTs had very low binding affinity for lysates overexpressing His_6_-tagged proteasome compared to those overexpressing Avi-tagged proteasome (Fig. 5c). Based on the on-grid purification setup, we could reconstruct the density map of 20S proteasome from cell lysates overexpressing Avi-tagged 20S proteasome (Fig. 5d).

The on-grid purification results demonstrate the CNT grid’s ability to directly isolate target proteins from cell lysates. We then explored whether these CNT-captured proteins could serve as a platform to recruit interacting partners from cell lysates. As a proof of principle, we used streptavidin-activated CNT grids to capture Avi-tagged GroES, followed by incubation with cell lysates overexpressing GroEL. This process led to the formation of high-density, well-assembled GroES/GroEL complex particles along the CNTs, exhibiting various orientations (Fig. 5f, h). In contrast, control experiments involving direct incubation of streptavidin-activated CNTs with GroEL-overexpressing lysates yielded no detectable GroEL or GroES/GroEL complex particles on the CNTs (Fig. 5e, g). Together, these results demonstrate that affinity-functionalized CNTs enable effective on-grid purification and also exhibit great potential for on-grid assembly of protein complexes from cell lysates.

### CNTs as a biocompatible substrate for cell culture and in situ structural analysis

**Fig. 6:**
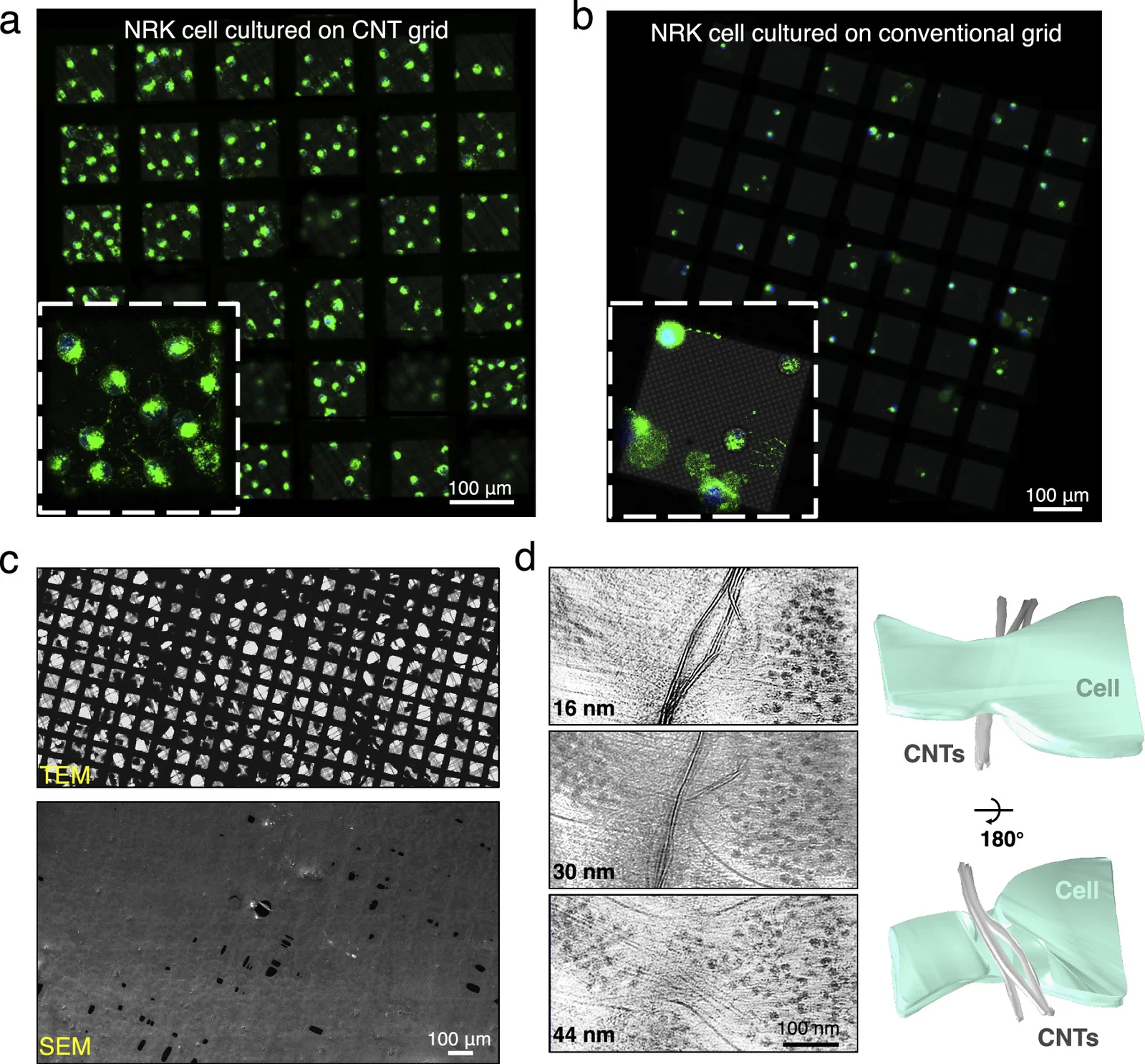
The application of CNT grids for in situ structural analysis. **a**,** b** Representative confocal microscopy images of NRK cells expressing GFP-tagged TSPAN7 (green) cultured for 4 h on a CNT grid (**a**) and a conventional holey carbon-coated EM grid (**b**). Insets provide detailed views of cell density and morphology. More than 3 grids were tested for each group independently with similar results. **c** A representative cryo-TEM image (top) and an SEM image (bottom) of NRK cells cultured on the CNT grid. More than 3 grids were tested independently with similar results. **d** Left: Three representative tomographic slices from the cryo-ET reconstruction of an NRK cell on a CNT grid. The relative height of each slice is indicated at the lower left, with the cryo-specimen boundary defined as the 0 nm plane. Right: The corresponding 3D segmentation model. More than 10 tilt-series were collected independently with similar results.

In addition to single-particle analysis, we also explored whether CNT grids could serve as a biocompatible substrate for in situ structural analysis of adherent cells (Fig. 6). To this end, we cultured Normal Rat Kidney Epithelial (NRK) cells on the CNT grids, where we observed dynamic cellular processes such as migration, retraction fiber and migrasome production^36^, as well as cell division events (Supplementary Fig. 14), confirming the grids’ suitability for live-cell imaging. In addition, we also observed that, under identical seeding conditions, cell density on CNT grids was ~5–6 times higher than on conventional EM grids (Fig. 6a, b and Supplementary Fig. 15). Moreover, after four hours of culturing, cells on CNTs exhibited a greater propensity to form retraction fibers, suggesting enhanced migratory activity and cell–cell communication. Long-term live-cell confocal imaging further revealed that cells on CNT grids were more motile than those on glass slides (Supplementary Videos 3, 4).

After culturing, cells on CNT grids were plunge-frozen for analysis by cryo‑TEM and cryo‑SEM (Fig. 6c). In the electron‑transparent region, we successfully identified and reconstructed both cellular structures and the CNT support (Fig. 6d and Supplementary Fig. 16). Furthermore, cryo‑FIB milling of cell bodies produced thin lamellae that enabled clear visualization of intracellular organelles (Supplementary Fig. 17). Collectively, these results establish CNT grids as a robust, cell-compatible platform for on-grid culture. We envision that with further optimization of the CNT network, this platform will advance integrated workflows in correlative light and electron microscopy (CLEM) and in situ cryo-EM structural biology.

## Discussion

This study presents the application of the CNT supporting film for cryo-EM specimen preparation. The CNTs, embedded within the vitreous ice, provide target macromolecules with greater freedom to interact in an omnidirectional configuration, which yields a diverse orientational distribution of particles. Various functionalization strategies of CNTs and their effects on the interactions between CNTs and target macromolecules were also systematically characterized in this work. CNTs modified with affinity ligands exhibit binding affinity and specificity to target proteins. Notably, the PEG_24_-biotin-modified CNT grid enables effective on-grid purification and/or assembly of target macromolecules, indicating promising potential for integrating sample purification and cryo-EM structural analysis. This capability also paves the way for structural investigations of biomolecules and their native interactors.

For the sample solution spreading characterization in the VitroJet setup, we observed that the hydrophilicity of the supporting film alone does not enable effective solution spreading on conventional EM grids (Fig. 2). To clarify this, we calculated the effect of hydrophilicity on liquid film formation, focusing on the final spreading state (Supplementary Fig. 18). In a localized region, such as a 100 μm square, a water contact angle as low as 5° (super-hydrophilic) would produce a liquid film thicker than 2 μm at its center. This result indicates that hydrophilicity alone is insufficient to achieve the desired liquid film thickness (e.g., sub-100 nm) for cryo-EM imaging. In contrast, CNTs, which generate significant capillary forces^37,38^, facilitate the spreading of the solution into a thin liquid film across the grid. This characteristic underscores the promising application of CNTs in blotting-free cryo-EM specimen preparation strategies.

The high binding specificity of affinity CNT grids enables the direct on-grid purification of target proteins from cell lysates, a capability that is not readily achievable with affinity graphene. We reason that the binding specificity of affinity graphene may be substantially lower than that of affinity CNTs. To test this, we compared PEG₂₄-biotin modified graphene and CNT grids for their binding specificity to Avi-tagged samples. We found PEG₂₄-biotin modified graphene captured target samples strongly even without prior streptavidin incubation, whereas after streptavidin activation, the target protein binding increased only slightly (Supplementary Fig. 19). In contrast, PEG₂₄-biotin modified CNTs displayed minimal binding to Avi-tagged samples unless first activated with streptavidin, after which they achieved high capture efficiency. These results indicate that graphene supports, even when functionalized with affinity ligands, exhibit substantially greater non-specific binding to macromolecules than do CNT grids. We attribute the selectivity differences to the intrinsic properties of the two materials. The planar structure of graphene maximizes the contact area and binding strength with all input proteins, promoting non-specific adsorption. In contrast, the highly curved, cylindrical surface of CNTs—especially those with small diameters—restricts the protein–surface contact area, thereby preventing stable adsorption unless the target proteins are specifically captured by the modifiers on the CNTs. This contrast highlights the greater potential of functionalized CNTs as a robust on-grid purification platform for cryo-EM structural analysis.

Substrate properties are known to influence the behavior and morphology of cultured cells^39–41^. We evaluated CNTs as a substrate for on-grid cell culture and in situ cryo-EM analysis. Under identical culture conditions, cell density was significantly higher on CNT grids than on conventional EM grids, confirming good biocompatibility. Interestingly, we observed a generally more uniform and intact ice layer on CNT grids than on conventional EM grids, likely due to the reinforced mechanical stability provided by the CNT–liquid composite^42^ (Supplementary Fig. 20). Together, these advantages position CNT grids as a valuable addition to the cryo-EM specimen preparation toolkit, applicable not only to isolated macromolecules but also to in situ cellular samples.

## Methods

### Preparation of the CNT grids

The super-aligned carbon nanotubes (SACNT) arrays were synthesized in batches using iron as the catalyst, which was deposited on an eight-inch silicon wafer substrate through low-pressure chemical vapor deposition (LPCVD)^43^. The growth temperature ranged from 600 to 800 °C, and acetylene was the carbon source gas. By varying the growth duration, SACNT arrays of different heights were obtained, with the heights of the arrays used here ranging from 300 to 800 μm.

A single-layer carbon nanotube (CNT) film was obtained from the SACNT arrays using the established procedure^28^. Specifically, a single layer of CNT film was drawn from the SACNT array using a plastic rod, and a second layer was drawn on top of it. These two layers of CNT films were then arranged in a vertically crossed pattern on a flat glass slide substrate (Supplementary Fig. 1). This substrate was later immersed in a container filled with deionized water, allowing the crossed CNT films to float on the water’s surface. A copper frame coated with adhesive was then pressed onto the floating CNT films and carefully lifted out of the water. Any remaining water droplets on the CNT film surface were gently absorbed using filter paper. To make the density of CNTs proper for TEM, the CNT films were pre-strained as required before being transferred onto EM grids. To adhere the crossed CNT films onto EM grids (Gilder grids, G300-G3), we first applied a small droplet of ethanol solution onto the EM grid surface. Excess ethanol was removed using filter paper, and then the grid was placed face-down onto the CNT films. A commercial YAG fiber laser cutting equipment, with a wavelength of 1.06 μm, was used to cut the CNT films at a laser speed of 1.5 m/s and with the laser power set to ~20 W (as shown in Supplementary Fig. 1). Finally, the CNT-coated EM grids were stored at room temperature in a dry environment.

### Characterization of the CNT grids

The transmittance of grids coated with CNT networks—prepared with varying pre-strain parameters—was measured using a Lambda 950 UV/VIS spectrometer (PerkinElmer) at a wavelength of 550 nm. The mechanical strength and Young’s modulus of CNT films (5 mm × 10 mm × 40 nm) produced under different pre-strain conditions were measured using a MicroTester 5848 (Instron).

Water contact angles were measured with an OCA15Pro contact angle measurement system (Dataphysics). CNT grids were subjected to various plasma cleaning durations (0 ~ 60 s) under an oxygen gas flow of 5 sccm in a plasma cleaning system (Gatan Solarus), and then were applied with a 1.5 μl deionized water droplet. At least five replicates were performed for each plasma cleaning duration.

VitroJet was used to observe the solution spreading process on both CNT grids and holey carbon-coated EM grids. Both grids were subjected to a duration of 40 s plasma cleaning before being applied with a droplet; the frame rate was 10 frames per second.

### Synthesis of diazonium salts

We added 0.056 g tert-butyl (4-aminobenzyl) carbamate (CAS 94838-55-8) to a 2 ml DMF solution containing 0.2 g Biotin-PEG_24_-NHS. The mixture was stirred at 80 °C for 12 h and then concentrated by vacuum distillation. The product was purified using rapid chromatography. The purified product was dissolved in 2 ml of dichloromethane and subsequently mixed with 4 M HCl in dioxane. The mixture was stirred at room temperature for 2 h and then concentrated under reduced pressure. The concentrated compound was dissolved in water and treated with 0.2 ml of 50% HBF_4_, followed by stirring at 0 °C for 5 min. Next, 1 g of Sodium Nitrite dissolved in 0.2 ml of deionized water was added. The mixture was stirred vigorously for 1 h and then filtered to produce a crude biotin-polyethylene glycol-aryl tetrafluoroborate diazonium salt solution.

### Functionalization of the CNT grids

For Ni^2+^-NTA functionalization, the CNT grid was subjected to a glow discharge for 40 s under an oxygen gas flow of 5 sccm in a plasma cleaning system (Gatan Solarus). Subsequently, 10 μl of 100 mM MES buffer (pH 6.0) containing 2 mM 1-ethyl-3-(3-dimethylaminopropyl) carbodiimide hydrochloride (EDC, Thermo Scientific Company, CAS 22980) and 5 mM N-hydroxyl-sulfosuccinimide (sulfo-NHS, Thermo Scientific Company, CAS 24525) was immediately pipetted onto the freshly glow-discharged grid. After incubation for 30 min at room temperature, the CNT grid was washed three times with 100 mM PBS buffer (pH 7.4) and activated with 10 μl PBS buffer containing 11.3 mM (S)-N-(5-Amino-1-carboxypentyl) iminodiacetic acid (NH_2_-NTA, Sigma-Aldrich Company, CAS 941689-36-7) for 2 h. The grid was then incubated with 11.3 mM NiSO_4_ solution for 30 min before being washed with deionized water or buffer for further characterization or sample loading. For NTA functionalization, the incubation with 11.3 mM NiSO_4_ solution was omitted, maintaining all other procedures and parameters as previously described.

For PEG_24_-biotin functionalization, the CNT grid was subjected to a glow discharge for 10 s under an argon gas flow of 5 sccm in a plasma cleaning system (Gatan Solarus). Subsequently, 3 μl of ionic liquid 1-butyl-3-methylimidazolium methyl sulfate (BMIMMeSO4, Macklin company, CAS 401788-98-5) containing 2 mM diazonium PEG_24_-biotin was pipetted onto the freshly glow-discharged grid. After incubation for 30 min at 37 °C, the CNT grid was washed twice with 1 ml acetonitrile and then immersed in deionized water for 10 min to ensure the complete removal of residual diazonium reagent.

### Functionalization of the graphene grids

For PEG_24_-biotin functionalization, the graphene grids were prepared by following previous protocols^31^. Graphene was grown on copper foil by the chemical vapor deposition (CVD) method. We firstly cut a piece of graphene-coated copper foil, which was then subjected to a glow discharge for 10 s under an argon gas flow of 5 sccm in a plasma cleaning system (Gatan Solarus), and dropped 2 mM diazonium PEG_24_-biotin salt solution onto the graphene surface, followed by a 30-min incubation at room temperature. Afterwards, the graphene membrane was washed twice with 1 ml acetonitrile and then immersed in deionized water for 10 min to ensure the complete removal of residual diazonium reagent. Then, we transferred the functionalized graphene membrane to holey carbon-coated EM grids using paraffin as the transfer medium.

### Characterization of the functionalized CNT grids

X-ray photoelectron spectra were collected using an ESCALAB 250XI (ThermoFisher Scientific), with the monochromatic Al Kα X-ray beam spot of 500 μm. Raman spectra were measured using a LabRAM HR-800 (Horiba) with a 532 nm laser wavelength.

### Preparation of biological samples

*Thermoplasma acidophilum* 20S proteasome with a C-terminal 6×His tag on the β subunit (termed as His_6_-tagged 20S proteasome) or an N-terminal Avi tag on the α subunit (termed as Avi-tagged 20S proteasome) was expressed and purified from *Escherichia coli* BL21 cells^32^. Briefly, the His_6_-tagged 20S proteasome was purified by nickel affinity column (GE Healthcare) followed by size exclusion chromatography on a Superose 6 10/300 column (Cytiva), and diluted to 0.3 mg/ml for use. The Avi-tagged 20S proteasome was purified using a strep-Tactin®XT column, followed by size exclusion chromatography on a Superose 6 10/300 column (Cytiva), and also diluted to 0.3 mg/ml for use. N-terminal Avi-tagged GroES and N-terminal Histidine- (6×His-) tagged GroEL were expressed in *Escherichia coli* BL21 cells^44^, purified using a Source Q column (Cytiva), and then subjected to size exclusion chromatography on a Superose 200 10/300 column (Cytiva). N-terminal Histidine- (6×His-) tagged GroEL was purified using a nickel affinity column (GE Healthcare) followed by size exclusion chromatography on a Superose 6 10/300 column (Cytiva). The GroES/GroEL complex was obtained by incubating GroES and GroEL at a molar ratio of 1:2 in a buffer containing 3 mM ATP, 10 mM MgCl₂, 100 mM KCl, and 1 mM BeF₂ for 20 minutes at room temperature, followed by further purification via size-exclusion chromatography using a Superose 6 5/150 column (Cytiva). Streptavidin was purchased from NEB (Catalog: N7021S).

To prepare the cell lysates used for on-grid purification, His_6_-tagged 20S proteasome, Avi-tagged 20S proteasome, and GroEL were all overexpressed from *Escherichia coli* BL21 cells. The cell pellet was resuspended in sample resuspension buffer (25 mM Tris-HCl, pH 8.0, 150 mM NaCl, 10% glycerol) at a ratio of 10 ml buffer per gram of wet cell weight. Following resuspension, phenylmethylsulfonyl fluoride (PMSF) was added to a final concentration of 1 mM (from a 100 mM stock in anhydrous ethanol) for protease inhibition. The suspension was then subjected to sonication on ice. The lysate was centrifuged at 15,000 × *g* for 1 h at 4 °C. The supernatant was collected for subsequent use.

### Cell culturing and fluorescence imaging

The NRK cell line stably expressing Tspan7-GFP was obtained from the laboratory of Prof. Li Yu at Tsinghua University. The cells were cultured at 37 °C with 5% CO_2_ in DMEM high glucose medium supplemented with 10% fetal bovine serum, 1% penicillin-streptomycin, and 1% GlutaMax.

For cell culturing on the CNT grids and conventional holey carbon-coated grids, the grids were placed in a 35-mm confocal dish and sterilized by exposure to ultraviolet light for 10 min. A solution of human plasma fibronectin (FC010, Millipore) at a concentration of 10 μg/ml in PBS buffer was then applied to the grids and incubated for 40 min at 37 °C. After incubation, the fibronectin solution was removed. NRK cells (1.2 × 10^5^ cells/ml) were seeded onto the CNT grids and cultured in an incubator at 37 °C with 5% CO_2_. For long-term time-lapse experiments, the cells were allowed to migrate for ~4 or 13 h before being transferred to a Nikon A1R HD25 confocal microscope equipped with a live cell imaging system. Images were acquired using a 60× objective at 1024 × 1024 pixels with a 2-minute interval over specified time periods and a 1 μm step size for the indicated Z distances. Atlas montage images were acquired using a 100× objective at a resolution of 1024 × 1024 pixels, with tiled acquisition in either 6 × 6 or 5 × 7 configurations. Z-stack images were subsequently processed by maximum-intensity projection. To observe cellular behavior on the glass slide versus on CNT grids at varying heights within the same confocal dish, long-term time-lapse z-stack imaging was performed. Prior to imaging, cells were allowed to migrate for ~12 h. Images were acquired at 20× magnification with a resolution of 1024 × 1024 pixels, at 16.5-min intervals for the indicated durations, using a z-step size of 1.13 μm.

### Preparation of cryo-EM specimen

For the non-functionalized CNT grid, a 3 μl sample solution containing 0.3 mg/ml His_6_-tagged 20S proteasome was pipetted onto the grid, which had been previously glow-discharged. The grid was then transferred into a Vitrobot Mark Ⅳ (ThermoFisher Scientific). The temperature of the Vitrobot chamber was set to 8 °C with a humidity of 100%, and the blotting time was set to 2 s with a force of −2. The grid was blotted with filter papers and then plunged into liquid ethane and stored in liquid nitrogen for cryo-EM imaging. For the conventional holey carbon-coated grid (Quantifoil, R1.2/1.3, 300 mesh), the concentration of 20S proteasome was 1 mg/ml, with other parameters and procedures the same as aforementioned.

For the Ni^2+^-NTA-modified CNT grid, a freshly prepared grid was incubated with 3 μl sample solution containing 0.3 mg/ml His_6_-tagged 20S proteasome. After incubation at 4 °C for 30 min, the grid was manually blotted from the back side and incubated with 3 μl buffer (50 mM Tris-HCl, 500 mM NaCl, pH 7.5) or 500 mM imidazole for an additional 5 min, before being transferred into the Vitrobot for subsequent procedures. For the NTA-modified CNT grid, gird was incubated with 3 μl buffer before being transferred into the Vitrobot, maintaining all other procedures and parameters as previously described.

For the specific binding of 20S proteasome with PEG_24_-biotin-modified CNT grid, the prepared grid was first incubated with 2 μl of 0.1 mg/ml streptavidin sample at 4 °C for 20 min. The grid was then washed with PBS buffer to remove excess streptavidin. Next, the grid was incubated with a 3 μl sample solution containing 0.3 mg/ml His-tagged 20S proteasome or 0.3 mg/ml Avi-tagged 20S proteasome at 4 °C for 30 min. After incubation, the grid was washed again with buffer (25 mM Tris-HCl, 500 mM NaCl, pH 8.0) to remove non-specific binding proteins. Finally, the grid was transferred into the Vitrobot for subsequent procedures, maintaining all parameters as previously described.

For the specific binding of Avi-tagged GroES with PEG_24_-biotin-modified CNT grid, the prepared grid was sequentially incubated with 0.1 mg/ml streptavidin and 0.75 mg/ml Avi-tagged GroES. Subsequently, to reconstitute the GroES/GroEL complex, the CNT grid was incubated with 3 μl of 3.8 mg/ml GroEL in a buffer containing 3 mM ATP, 10 mM MgCl_2_, 100 mM KCl, and 1 mM BeF_2_ for 1 h. Finally, the grid was washed with buffer (25 mM Tris-HCl, 500 mM NaCl, 3 mM ATP, 10 mM MgCl_2_, 100 mM KCl, 1 mM BeF_2_, pH 8.0) to remove non-specific binding proteins. Finally, the grid was transferred into the Vitrobot for subsequent procedures, maintaining all parameters as previously described. For the interactions between particles and PEG_24_-biotin-modified CNTs, 4.13 mg/ml GroES/GroEL complex was incubated with PEG_24_-biotin-modified CNT grid for 30 min at 4 °C. Following incubation, the grid was processed using two protocols: one was directly transferred into the Vitrobot for subsequent procedures, maintaining all parameters as previously described; the other was washed with buffer (25 mM Tris-HCl, 500 mM NaCl, 3 mM ATP, 10 mM MgCl_2_, 100 mM KCl, 1 mM BeF_2_, pH 8.0). Subsequently, the washed grid was plunge-frozen using the Vitrobot under the same standardized conditions.

For on-grid purification of 20S proteasome from cell lysates, the PEG_24_-biotin-modified CNT grid was sequentially incubated with 0.1 mg/ml streptavidin and then cell lysates containing overexpressed Avi-tagged 20S proteasome for 1 h at 4 °C, and washed with buffer (25 mM Tris-HCl, 500 mM NaCl, pH 8.0) three times. Then the grid was transferred into the Vitrobot for subsequent procedures, maintaining all parameters as previously described.

For on-grid purification of GroEL from cell lysates and assembly on CNT grid, the PEG_24_-biotin-modified CNT grid was sequentially incubated with 0.1 mg/ml streptavidin and then 0.75 mg/ml Avi-tagged GroES, and washed with buffer (25 mM Tris-HCl, 500 mM NaCl, 3 mM ATP, 10 mM MgCl_2_, 100 mM KCl, 1 mM BeF_2_, pH 8.0). The grid was then immersed in cell lysates containing overexpressed GroEL at room temperature for 1 h and finally washed with buffer (25 mM Tris-HCl, 500 mM NaCl, 3 mM ATP,10 mM MgCl_2_, 100 mM KCl, 1 mM BeF_2_, pH 8.0) three times before plunge freezing.

For the cryo-EM specimen preparation of cell samples on grids, CNT grids or conventional holey carbon-coated grids bearing adherent cells were gently retrieved from the confocal dish using tweezers after 14–16 h of culturing, and 5 μl cell culture medium was applied to each grid. The grids were then transferred to a Leica EM GP for cryo-EM specimen preparation. The Leica EM GP was operated at 37 °C and 90% humidity, with a blotting time of 8–10 s. Grids were blotted from the back side and immediately plunge-frozen into liquid ethane. Finally, the vitrified grids were stored in liquid nitrogen until loading for FIB milling or cryo-EM imaging.

### Silver staining SDS-PAGE

For silver staining, each grid was immersed in a tube containing 10 μl of 1× SDS-PAGE loading buffer (Beyotime) and incubated at 98 °C for 20 min. The incubated solution was resolved on sodium dodecyl sulfate-polyacrylamide gels (SDS-PAGE) and subjected to silver staining. The silver staining signal was detected using a Silver Stain Kit (Beyotime) and visualized by a ChemiDoc MP Imaging system (Bio-Rad, ChemiDoc MP) for further analysis.

For the comparison of PEG_24_-biotin-modified graphene and CNT grids regarding their passivated interface for resisting non-specific adsorption and their specific capture capability, each grid was incubated with 3 μl of buffer containing 1 μg Avi-tagged 20S proteasome for 30 min, followed by three washes with buffer before undergoing the silver staining SDS-PAGE experiments.

The uncropped gel images for Fig. 5a, b, e and f are provided in the Source Data file, and the uncropped gel images for Fig. S11a and S19 are provided in the Supplementary Information file.

### Cryo-EM data collection and structural analysis

The Cryo-EM single-particle dataset of non-functionalized CNTs grid for 20S proteasome was automatically collected using EPU software on a Thermo Fisher Scientific Titan Krios G3i TEM (300 kV), equipped with a GIF-Quantum energy filter (20 eV), a Gatan K3 direct electron detector, and a Cs-corrector. A total of 1,205 micrographs were collected with a pixel size of 1.08 Å. For the dataset of PEG_24_-biotin-modified CNTs grid for 20S proteasome from cell lysates, GroES/GroEL complex and GroES/GroEL complex from cell lysates, 1023, 1032, and 1149 micrographs were automatically collected, respectively, with a pixel size of 1.0742 Å, using AutoEMation software^45^ on a Thermo Fisher Scientific Titan Krios TEM (300 kV), equipped with a K3 camera (Gatan, Inc.) and a GIF-Quantum energy filter (20 eV). For the 20S proteasome dataset on holey carbon-coated grid and GroES/GroEL complex dataset on the open region of CNT grid, 91 and 660 micrographs with a pixel size of 0.836 Å were automatically collected, respectively, using SerialEM software^46^ on an FEI Tecnai Arctica TEM (200 kV) equipped with a Gatan K2 camera.

During data collection, all the micrographs were fractioned to 32 frames with a total electron dose of 50 e^−^/Å^2^ and motion-corrected using MotionCor2^47^. The motion-corrected micrographs were imported into CryoSPARC for patch CTF estimation, micrograph selection, Topaz particle picking, 2D classification, and 3D homo-refinement^48^. In 2D classification, we disabled the “Re-center” option and applied a tight mask with a diameter slightly larger than that of the target particle. We found that this approach best eliminated CNT interference during alignment and yielded more usable particles for subsequent reconstruction. The numbers of particles used for the final 3D reconstruction were as follows: 60,241 for 20S proteasome on non-functionalized CNTs, 16,627 for 20S proteasome on holey carbon-coated grid, 18,946 for 20S proteasome from cell lysates on PEG_24_-biotin-modified CNTs (Supplementary Fig. 21), 14,551 for GroES/GroEL complex on PEG_24_-biotin-modified CNTs, 6140 for GroES/GroEL complex from cell lysates on PEG_24_-biotin-modified CNTs, and 19,064 for GroES/GroEL complex on open region of CNT grid. It’s worth noting that the particles used in the final reconstructions are distributed along the nanotubes and do not overlap with them. The reported resolutions were 2.8 Å, 3.1 Å, 3.1 Å, 3.4 Å, 4.1 Å, and 4.3 Å, respectively. Structure detail was visualized using Chimera^49^. The cryo-EM data collection and analysis are summarized in Supplementary Table 1. The Euler angles for each particle in the final particle dataset were plotted for the orientational distribution analysis.

### Cryo-FIB milling

Cryo-FIB milling was executed using a PFIB/SEM dual-beam system (Hydra, Thermo Fisher Scientific). Grids were clipped into autogrids under liquid nitrogen and transferred into the instrument chamber, where the pressure and temperature were maintained at ~3 × 10⁻⁷ mbar and −190 °C. For charge dissipation and sample protection, a three-step platinum coating protocol was employed: an initial sputter coating, followed by GIS deposition of organometallic platinum (60 s injection), and a final sputter coating step. The stage was tilted at 12°, corresponding to a milling angle of ~15° relative to the grid surface. Micro-expansion joints were milled to alleviate stress in the support film. Rough milling was performed at 30 kV, with the ion current being gradually decreased from 3.0 to 0.5 nA to produce lamellae of ~500 nm thickness. Fine milling and polishing were subsequently performed using currents of 50 and 30 pA, resulting in final lamellae with a thickness of 100–200 nm.

### Cryo-ET data collection and analysis

The Cryo-ET tilt series was collected using SerialEM^46^, with tilt angles ranging from +60° to −60° at 3° intervals, on a Thermo Fisher Scientific Titan Krios TEM (300 kV) equipped with a GIF-Quantum energy filter (20 eV) and a Gatan K3 direct electron detector. The pixel size for the His-tagged 20S proteasome sample and GroES/GroEL complex on the PEG_24_-biotin-modified CNTs grid was 1.359 Å, while for other samples, it was 1.2 Å. Each tilt angle contained 8 frames, with a summed dose of 3 e^−^/Å^2^, resulting in a total dose of 120 e^−^/Å^2^ for each tilt series (+60° to −60°). For the data acquisition of cell culturing on CNT grids, the pixel size was set as 2.56 Å, with a dose-symmetric automated data collection, covering an angular range of −51° to +51° (3° increments) and a defocus range of −4.5 to −5.5 μm. The tilt series were motion-corrected using MotionCor2^47^ and imported into IMOD^50^ for further alignment and reconstruction. The final tomograms were generated with a binning factor of 4.

### Reporting summary

Further information on research design is available in the Nature Portfolio Reporting Summary linked to this article.

## Supplementary information

Supplementary information

Description of Additional Supplementary File

Supplementary Video 1

Supplementary Video 2

Supplementary Video 3

Supplementary Video 4

Reporting Summary

Transparent Peer Review file

## Source data

Source data

## Data Availability

The cryo-EM density maps of GroES/GroEL on streptavidin-activated CNT, 20S proteasome from cell lysates on streptavidin-activated CNT and GroES/GroEL from cell lysates on streptavidin-activated CNT have been deposited into Electron Microscopy Data Bank (EMDB) under the accession numbers of EMD-80845, EMD-80844 and EMD-80843, respectively. The raw data of GroES/GroEL on streptavidin-activated CNT, 20S proteasome from cell lysates on streptavidin-activated CNT and GroES/GroEL from cell lysates on streptavidin-activated CNT have been deposited into the EMPIAR, with the corresponding accession codes EMPIAR-13560, EMPIAR-13561, and EMPIAR-13562, respectively. Source data are provided with this paper. The source data underlying Fig. 2a, 3a, 3c, d, 3f, 5a–c, 5e–g and Supplementary Fig. 2a, b, 4, 9a, b, and 10a–c are provided as a Source Data file. Source data are provided with this paper.
