# Supplementary material for: Carbon nanotubes as the supporting film for cryo-EM specimen preparation
Source: Nat Commun. 2026 Sep 21;17:11016. doi: 10.1038/s41467-026-77953-8 (PMC13686963; doi:10.1038/s41467-026-77953-8)

Supplementary Information

Carbon nanotubes as the supporting film for cryo-EM specimen preparation

Jiling Song[1,2,3†], Zhengni Wang[1,2,3†], Duanliang Zhou[4,5†], Zi Yuan[4,5*], Xia Jia[1,2,3,6], Xiaopeng Li[1,2,3,6], Chenhui Zhang[7], Yongbo Liu[3,6,8], Yu Rao[3,6,8], Dongju Wang[1,3], Kaili Jiang[4,5], Hong-Wei Wang[1,2,3,6], Nan Liu[7*]

[1]*School of Life Sciences, Tsinghua University, Beijing 100084, China.*

[2]*Ministry of Education Key Laboratory of Protein Sciences, Tsinghua University, Beijing 100084, China.*

[3]*Beijing Frontier Research Center for Biological Structures, Tsinghua University, Beijing 100084, China.*

[4]*State Key Laboratory of Low Dimensional Quantum Physics, Department of Physics, Tsinghua University, Beijing 100084, China.*

[5]*Frontier Science Center for Quantum Information, Department of Physics, Tsinghua University, Beijing 100084, China.*

[6]*Tsinghua-Peking Joint Center for Life Sciences, Tsinghua University, Beijing 100084, China.*

[7]*School of Biological Sciences, The University of Hong Kong, Hong Kong, 999077, China.*

[8]*School of Pharmaceutical Sciences, Tsinghua University, Beijing, 100084, China.*

*Correspondence should be addressed to Z.Y., or N.L.

(e-mail: yzkk@tsinghua.edu.cn or nanliu@hku.hk)

† These authors contributed equally to this work.

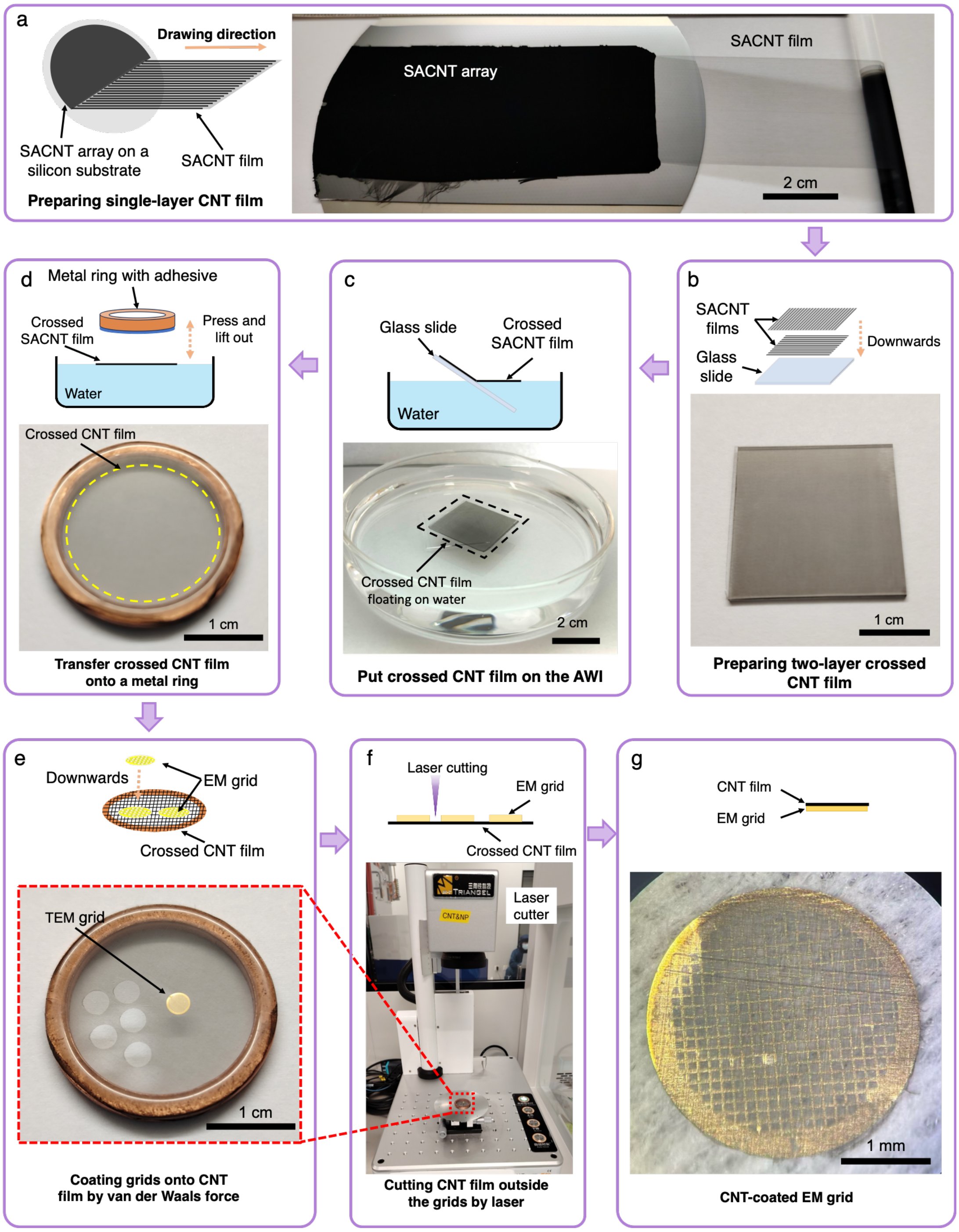

Supplementary Figure 1. Schematic of the preparation procedure of CNT grids. A single-layer CNT film was drawn from the SACNT arrays (**a**), followed by arranging a second layer of the CNT film onto the first CNT film in a vertically crossed pattern (**b**). The crossed CNT films were then floated on the water's surface (**c**), with a copper ring used to lift them out of the water (**d**). Subsequently, EM grids were placed onto the crossed CNT films (**e**), and a laser cutter was utilized to trim the excess CNTs outside the EM grids (**f**) to obtain the CNT-coated EM grids (**g**).

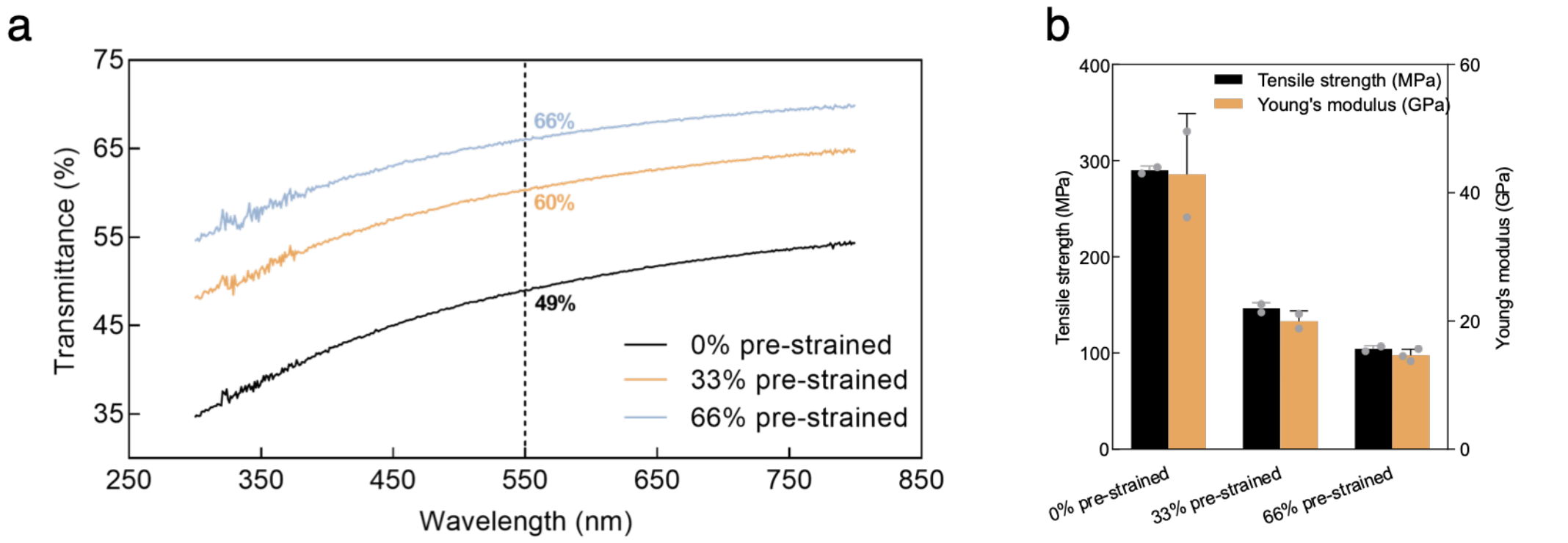

Supplementary Figure 2. Characterization of optical and mechanical properties of CNT films prepared with varying pre-strain. a, Transmittance at 550 nm of crossed CNT films with 0%, 33%, and 66% pre-strained, measured as 49%, 60%, and 66%, respectively. **b,** Tensile strength and Young's modulus of crossed CNT films with 0%, 33%, and 66% pre-strained. At least two grids were used to measure the tensile strength and Young's modulus for each condition. Source data are provided as a Source Data file.

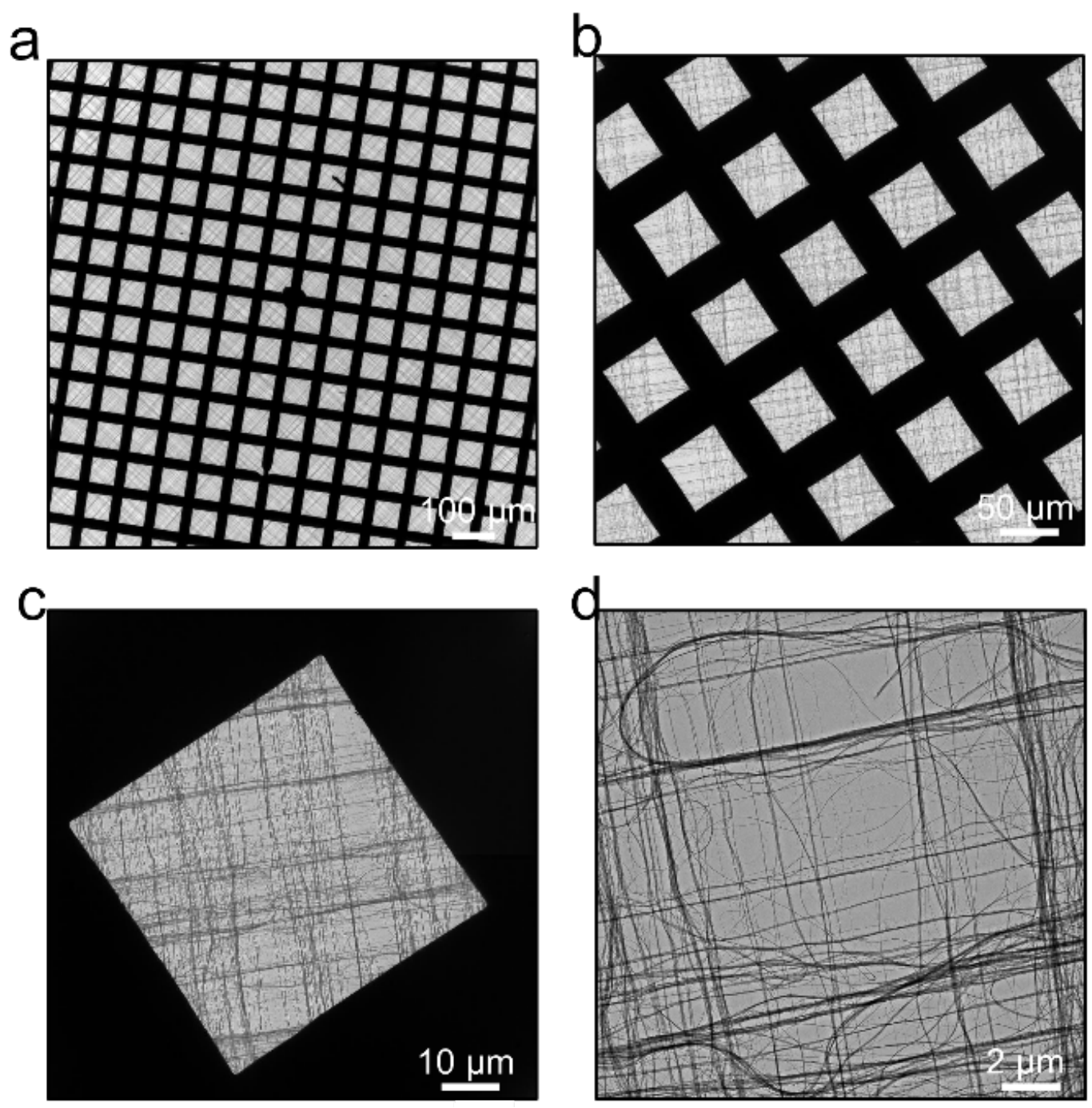

Supplementary Figure 3. TEM images under different magnifications of the CNT grid, with sequentially higher magnifications from (a) to (d). More than 100 micrographs were captured independently with similar results.

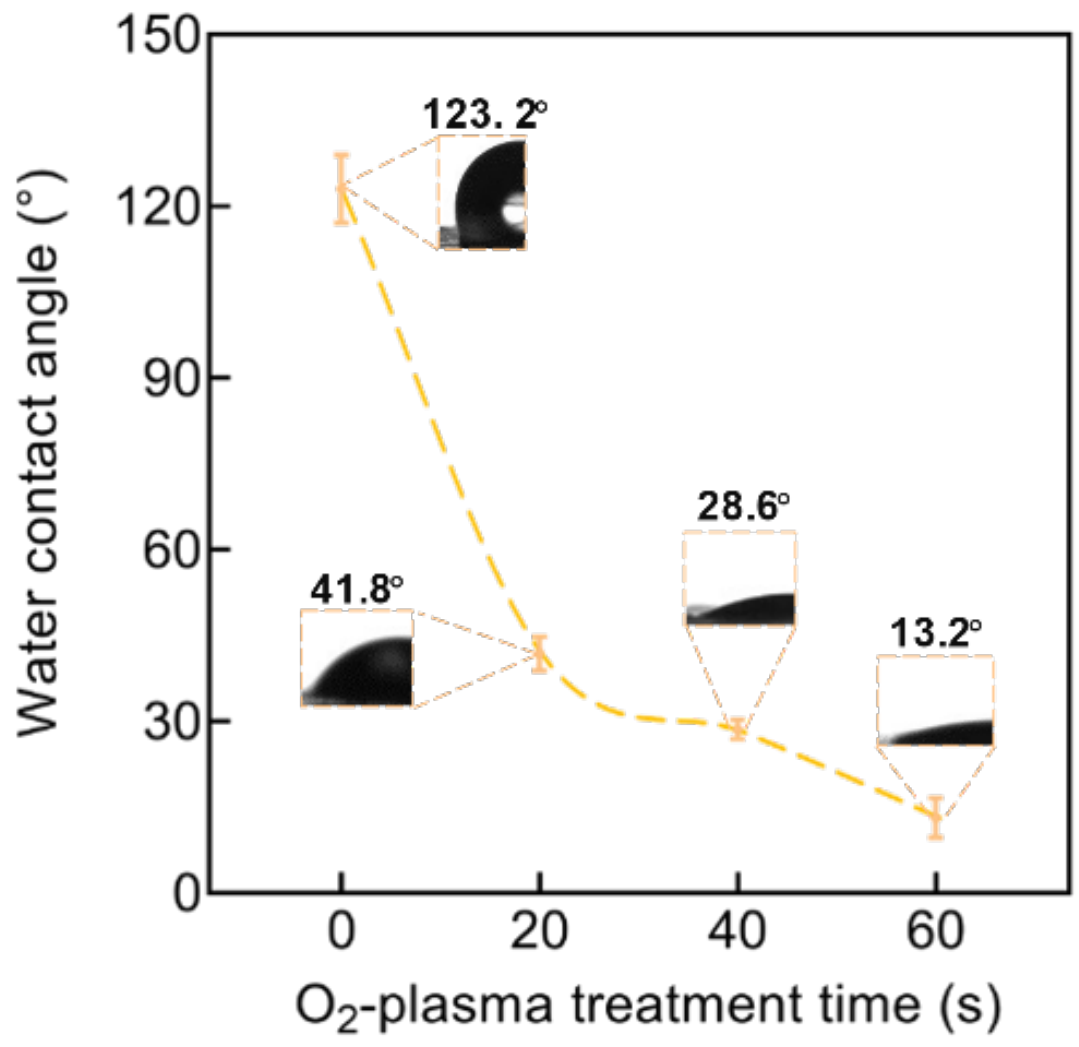

Supplementary Figure 4. Hydrophilicity of CNT grids after oxygen plasma treatment for different durations. Five grids were used to measure the water contact angles independently with similar results. Source data are provided as a Source Data file.

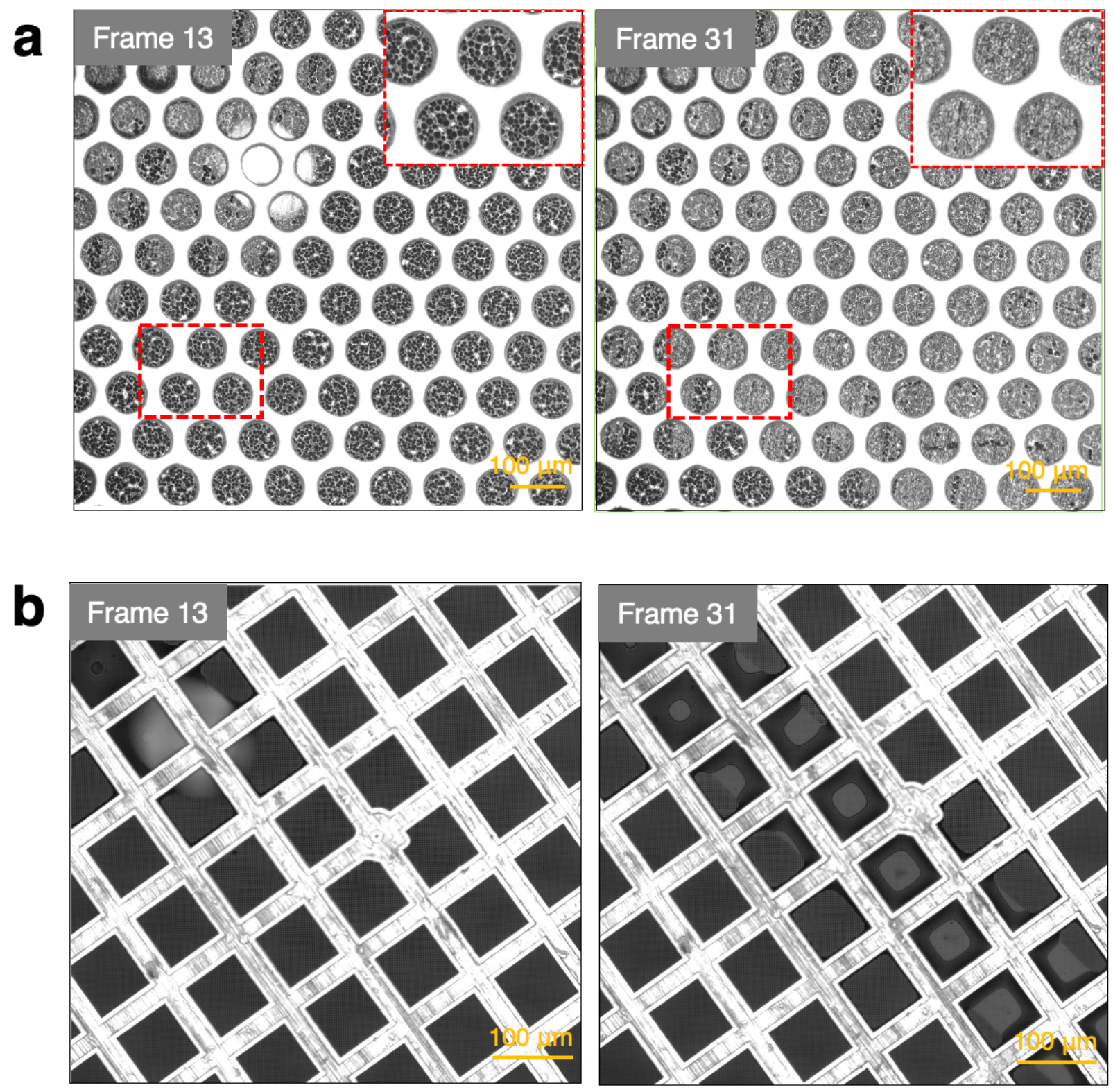

Supplementary Figure 5. Sequential frames (1.7 s apart) captured using VitroJet demonstrate fluid movement across a CNT grid (a) and a conventional holey carbon-coated EM grid (b). Three grids were tested for each group independently with similar results.

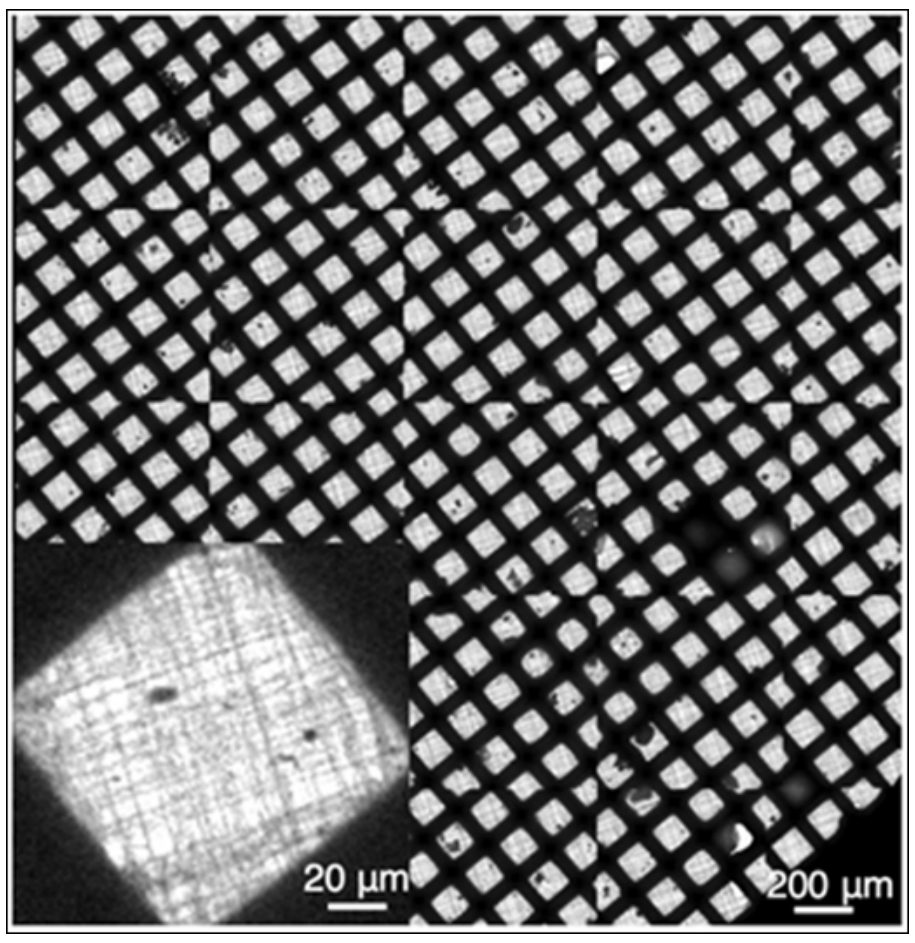

Supplementary Figure 6. Atlas of the cryo-EM specimen prepared with the CNT grid. More than 20 micrographs were captured independently with similar results.

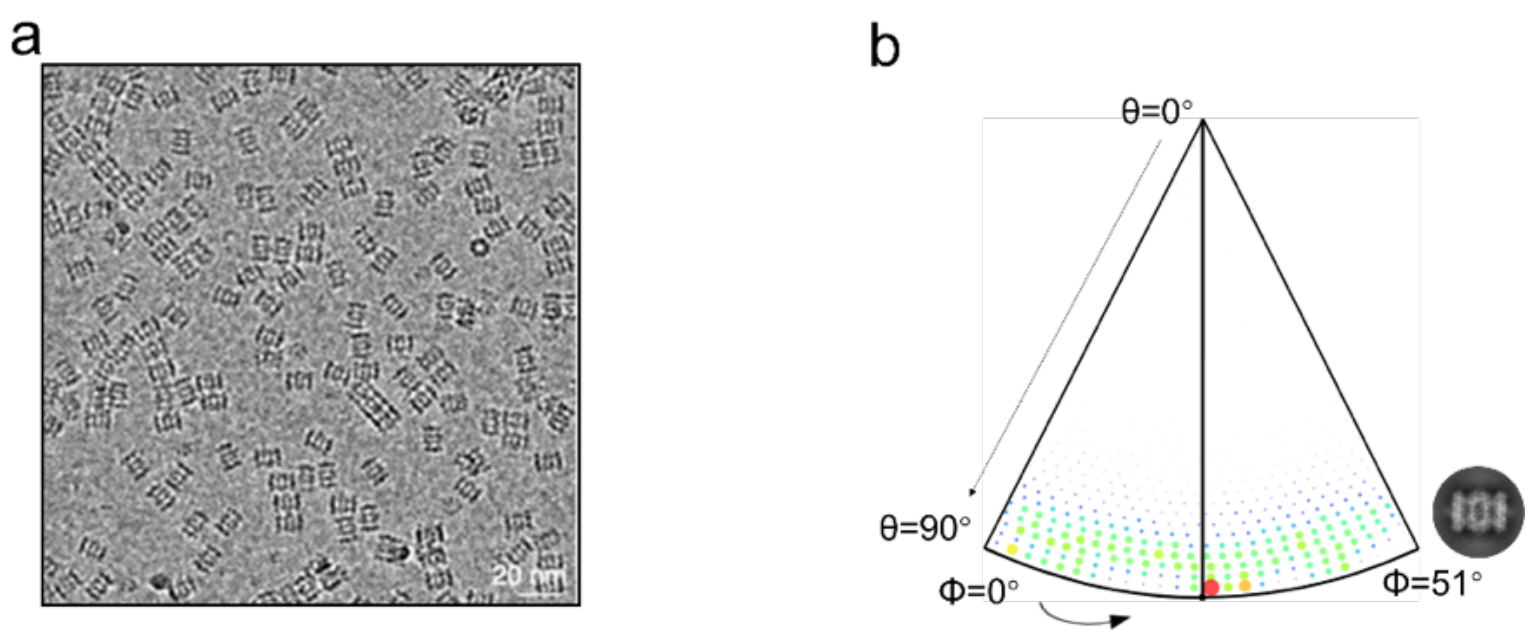

Supplementary Figure 7. Orientation analysis of 20S proteasome on a conventional holey carbon-coated EM grid. a, Representative cryo-EM micrograph of the 20S proteasome. More than 100 micrographs were captured independently with similar results. **b,** Angular distribution plot showing particle orientations on the conventional grid.

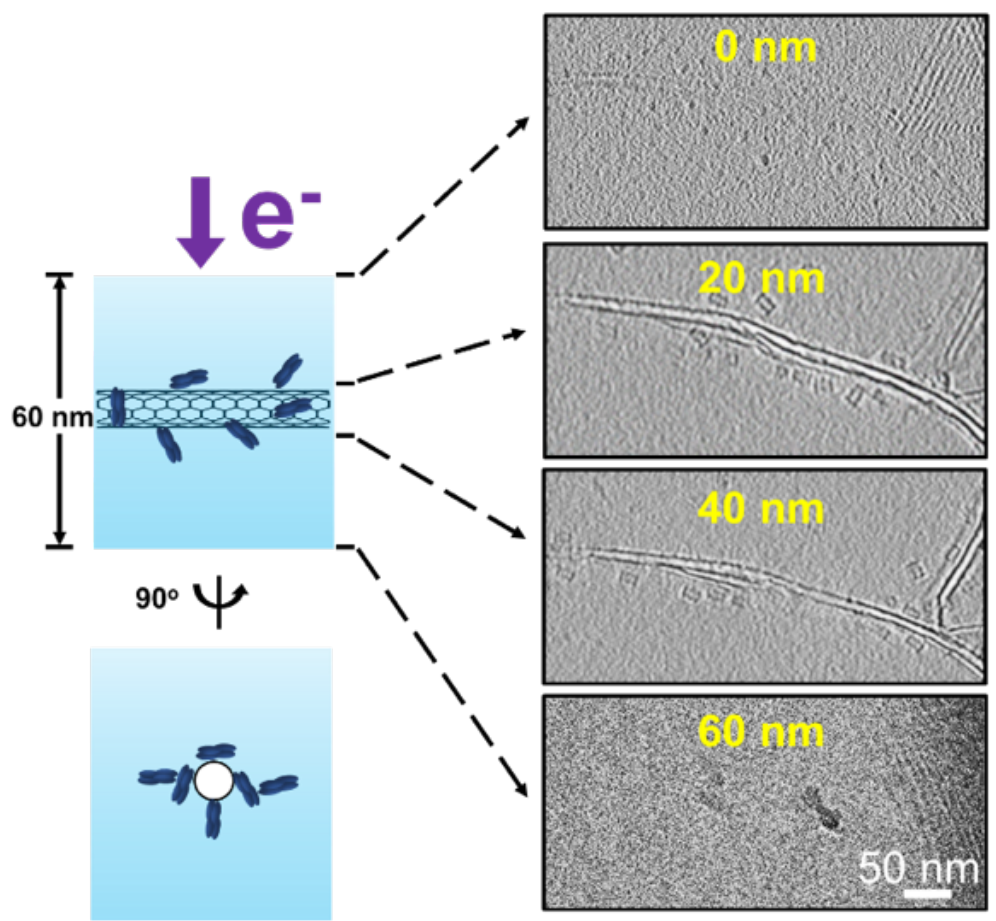

Supplementary Figure 8. Cryo-ET analysis of the CNT-supported specimen. Four representative slices through the Z-axis from the tomogram reconstruction are shown, with their relative locations indicated by arrows. The upper air-water interface is defined as 0 nm, and the

bottom air-water interface is 60 nm away from the upper air-water interface. More than 10 tilt-series were collected independently with similar results.

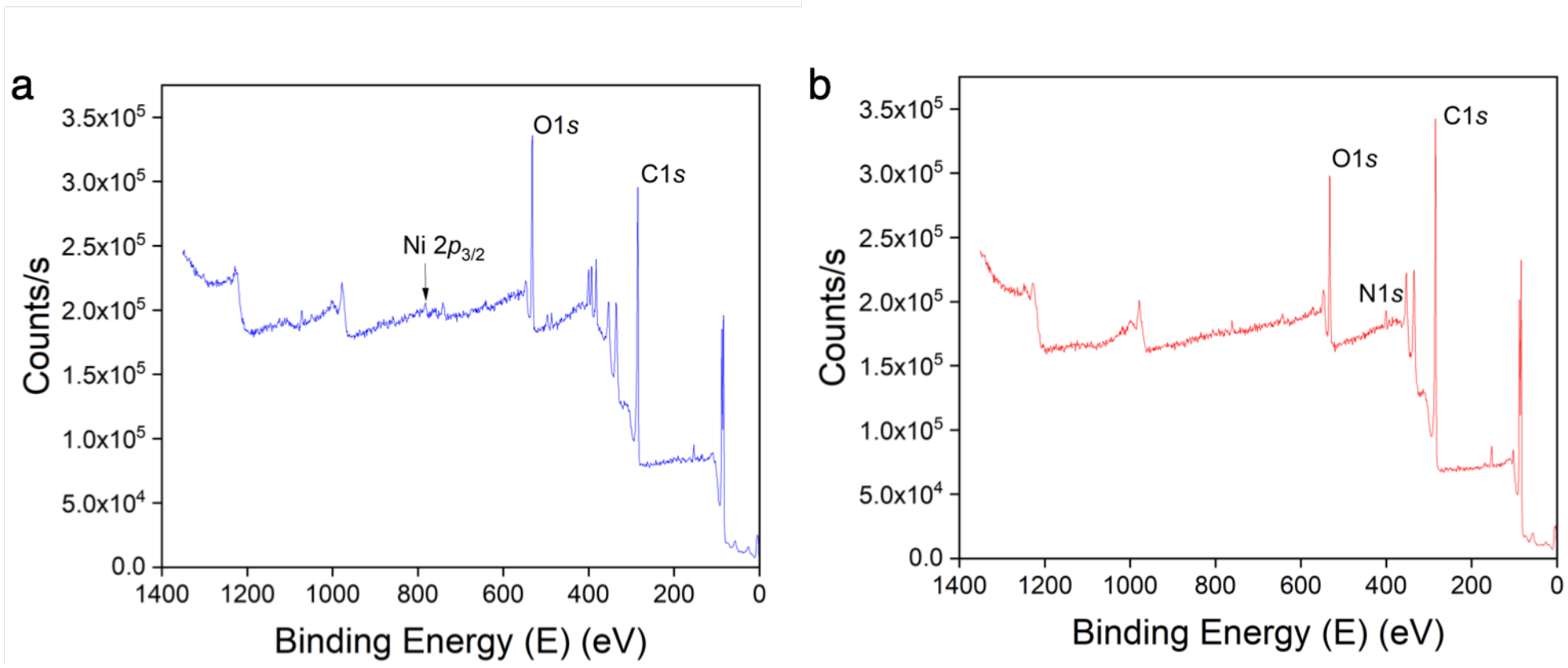

Supplementary Figure 9. X-ray photoelectron spectra of Ni^{2+}-NTA-modified CNTs (a) and PEG_{24}-biotin-modified CNTs (b). Source data are provided as a Source Data file.

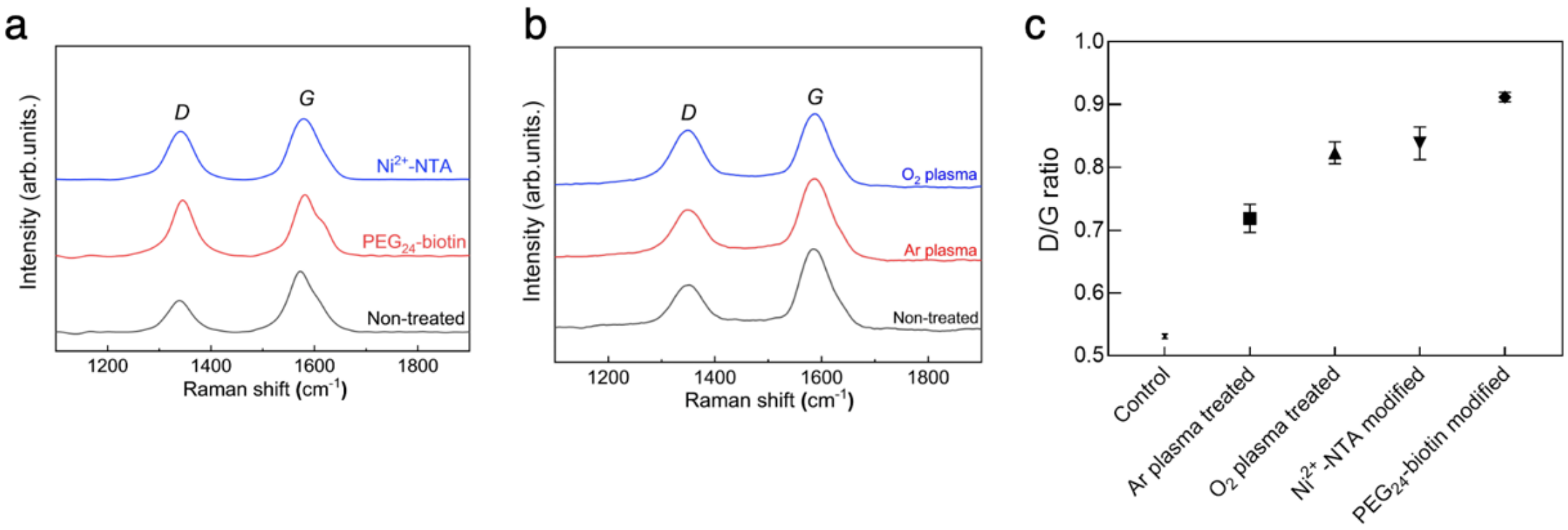

Supplementary Figure 10. Raman characterization of CNTs. a, Representative Raman spectra of non-treated CNTs (black), PEG_{24}-biotin-modified CNTs (red), and Ni^{2+}-NTA-modified CNTs (blue). **b,** Representative Raman spectra of non-treated CNTs (black), plasma-treated CNTs under Ar atmosphere (red) and O_2 atmosphere (blue). **c**, Average Raman *D*/*G* intensity ratios for non-treated CNTs (Control), plasma-treated CNTs under Ar atmosphere and O_2 atmosphere, Ni^{2+}-NTA-modified CNTs, and PEG_{24}-biotin-modified CNTs. The averaged ratios are 0.53, 0.72, 0.81, 0.83, and 0.91, respectively. For each condition, measurements were repeated 3 times. Source data are provided as a Source Data file.

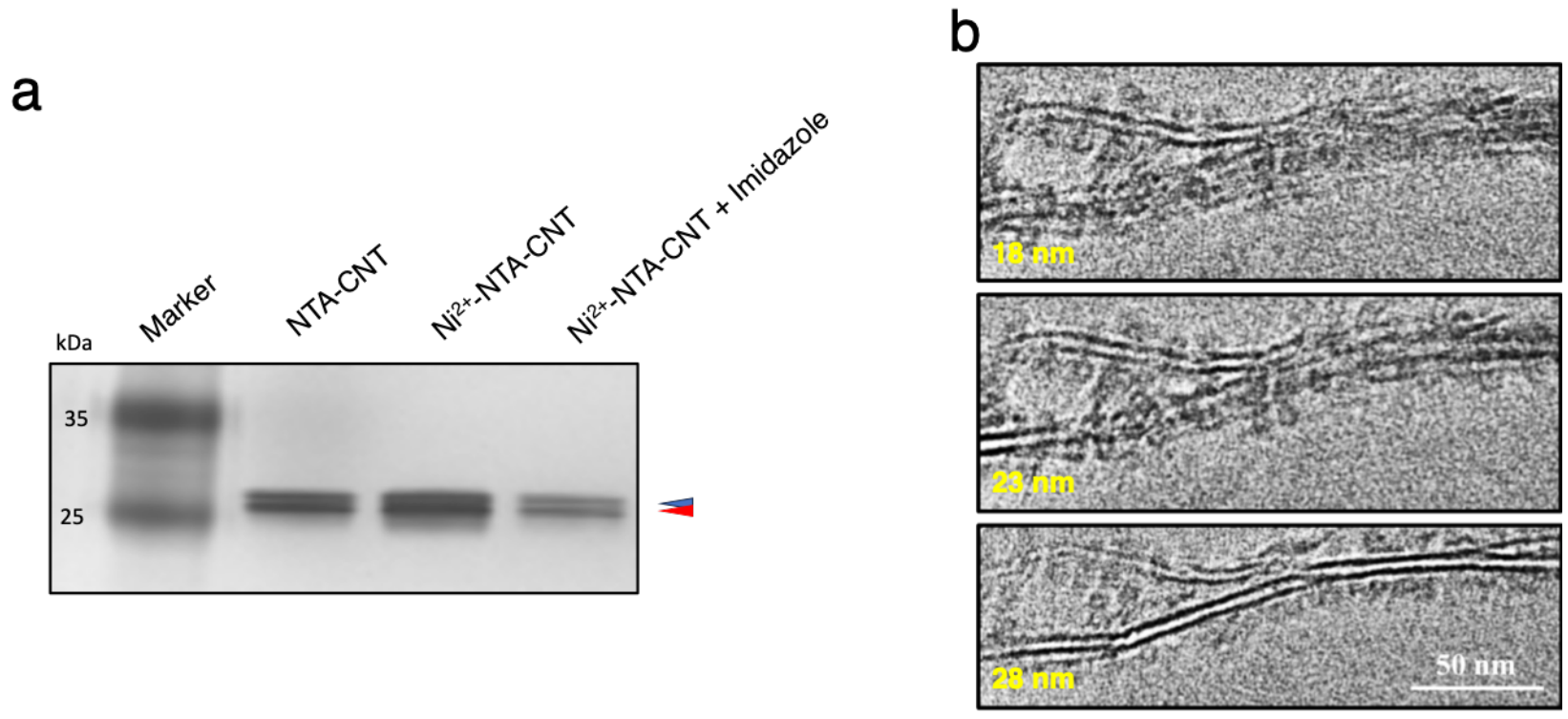

Supplementary Figure 11. Selective binding of Ni^{2+}-NTA-modified CNTs to 20S proteasome particles. a, Silver-stained SDS-PAGE gel. Lanes (left to right): molecular weight marker; NTA-modified CNTs incubated with His_6-tagged 20S proteasome; Ni^{2+}-NTA-modified CNTs incubated with His_6-tagged 20S proteasome; Ni^{2+}-NTA-modified CNTs incubated with His_6-tagged 20S proteasome followed by washing with imidazole. The α and β subunits of the 20S proteasome are indicated by blue and red arrows, respectively. Three experiments were repeated independently with similar results. **b**, Selected slices from cryo-ET reconstruction representing His_6-tagged 20S proteasome particles anchored on the Ni^{2+}-NTA modified CNTs. More than 10 tilt-series were collected independently with similar results. Source data are provided as a Source Data file.

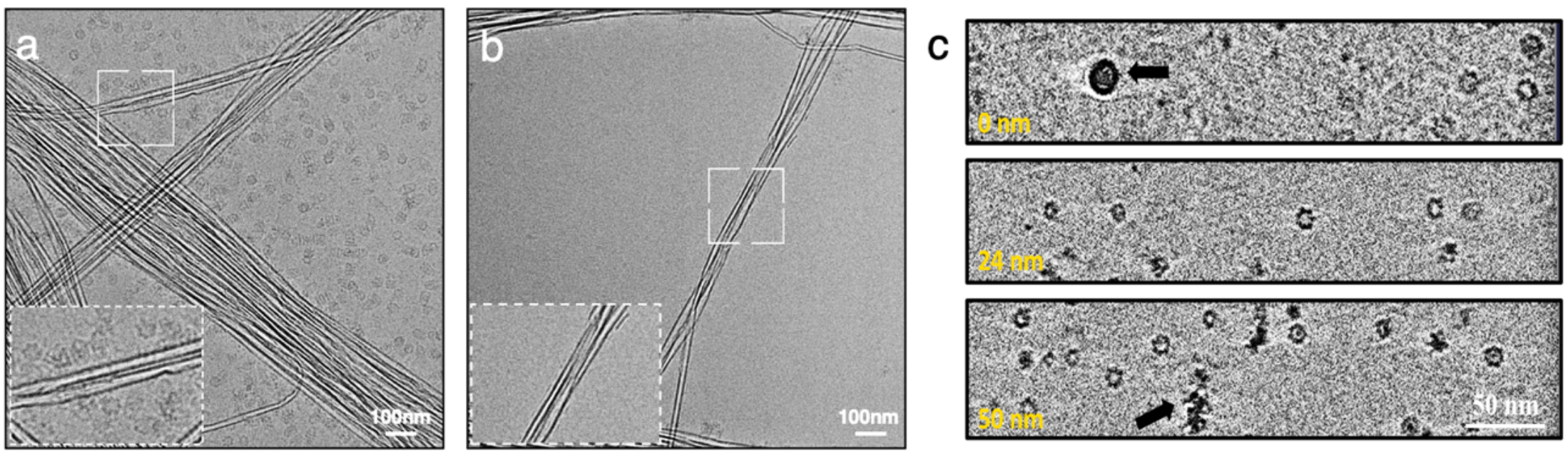

Supplementary Figure 12. Cryo-EM characterization of interactions between particles and the PEG_{24}-biotin-modified CNT grid. a-b, Representative cryo-EM images of (**a**) CNTs grid incubated with GroES/GroEL complex without wash and (**b**) PEG_{24}-biotin-modified CNTs incubated with GroES/GroEL complex and then washed with buffer. **c**, Selected tomographic slices illustrating particle distribution in open regions. The top slice corresponds to the upper boundary (0-nm reference layer). The middle slice corresponds to a central layer. The bottom slice corresponds to the lower boundary, located ~50 nm from the upper boundary. Ice contamination at the boundaries is indicated with black arrows. More than 100 micrographs and more10 tilt-series for each group were collected independently with similar results.

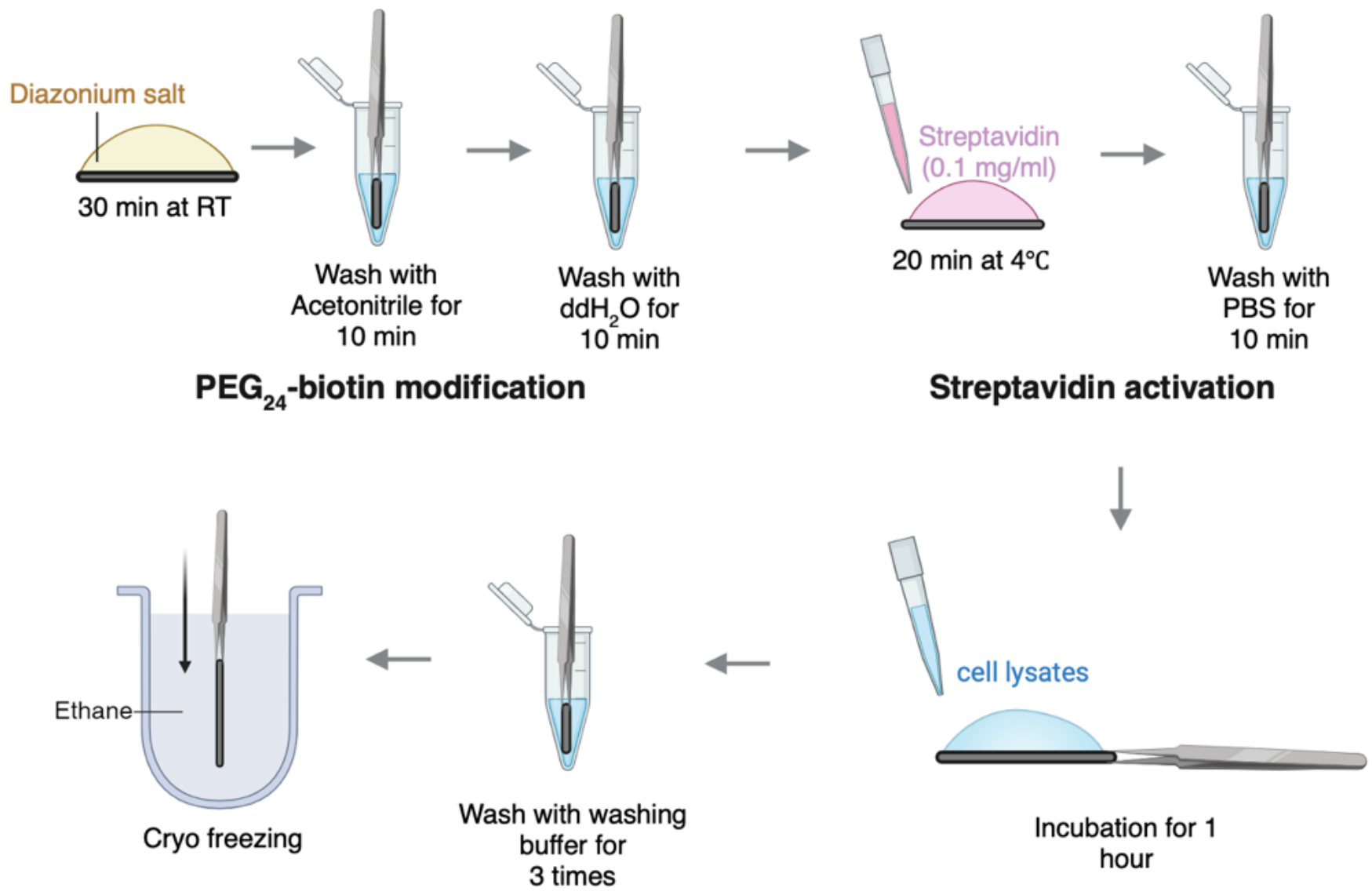

Supplementary Figure 13. Workflow of the on-grid purification or complex assembly using the PEG_{24}-biotin-modified CNT grid. This workflow was created with BioRender.com.

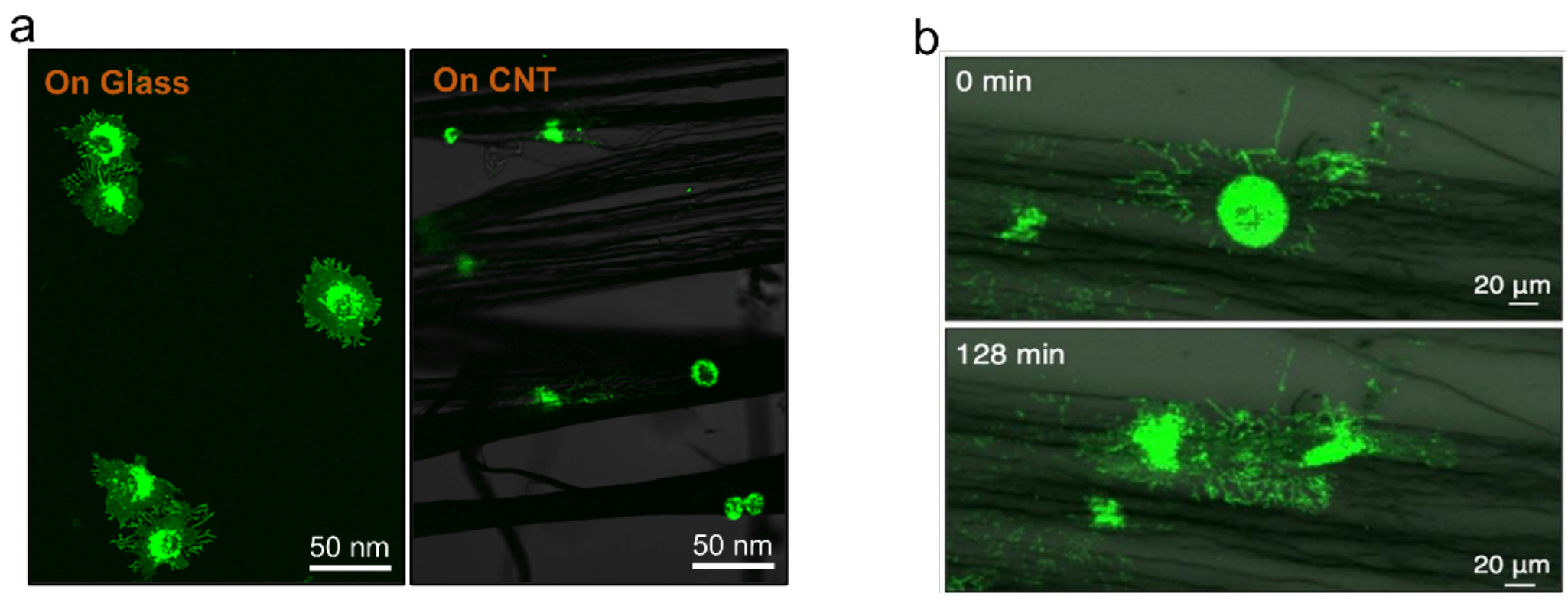

Supplementary Figure 14. Confocal microscopy of NRK cells cultured on the CNT grid. a, Representative confocal microscopy images of NRK cells cultured on a glass slide and on a CNT grid. **b**, Two time-lapse confocal microscopy frames capturing a cell division event on CNT support. More than 5 micrographs or videos for each group were collected independently with similar results.

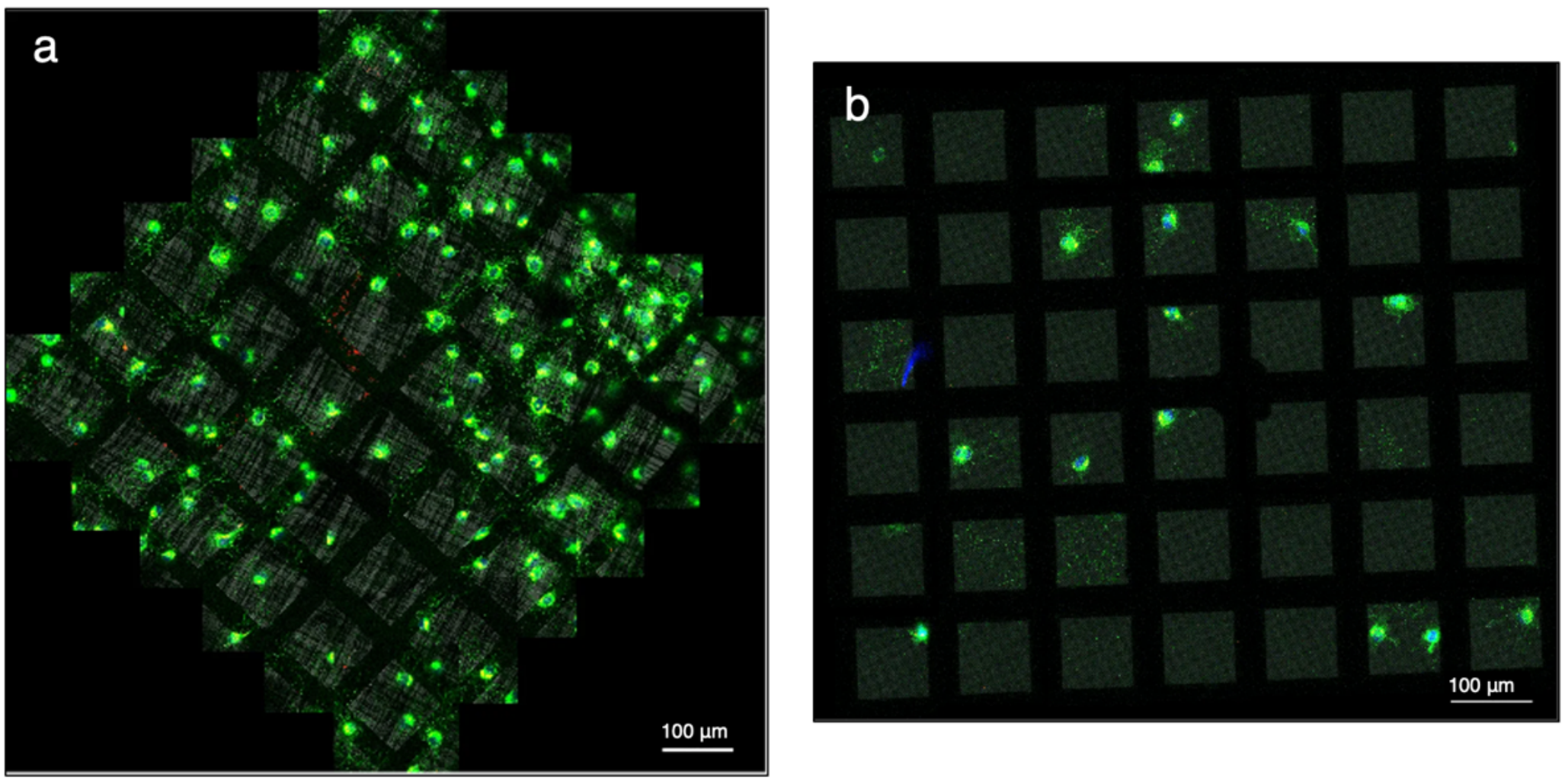

Supplementary Figure 15. Representative confocal images of NRK cells expressing GFP-tagged T7 protein (green) cultured for 13 hours on (a) a CNT grid and (b) a conventional holey carbon-coated EM grid. More than 3 grids were tested for each group independently with similar results.

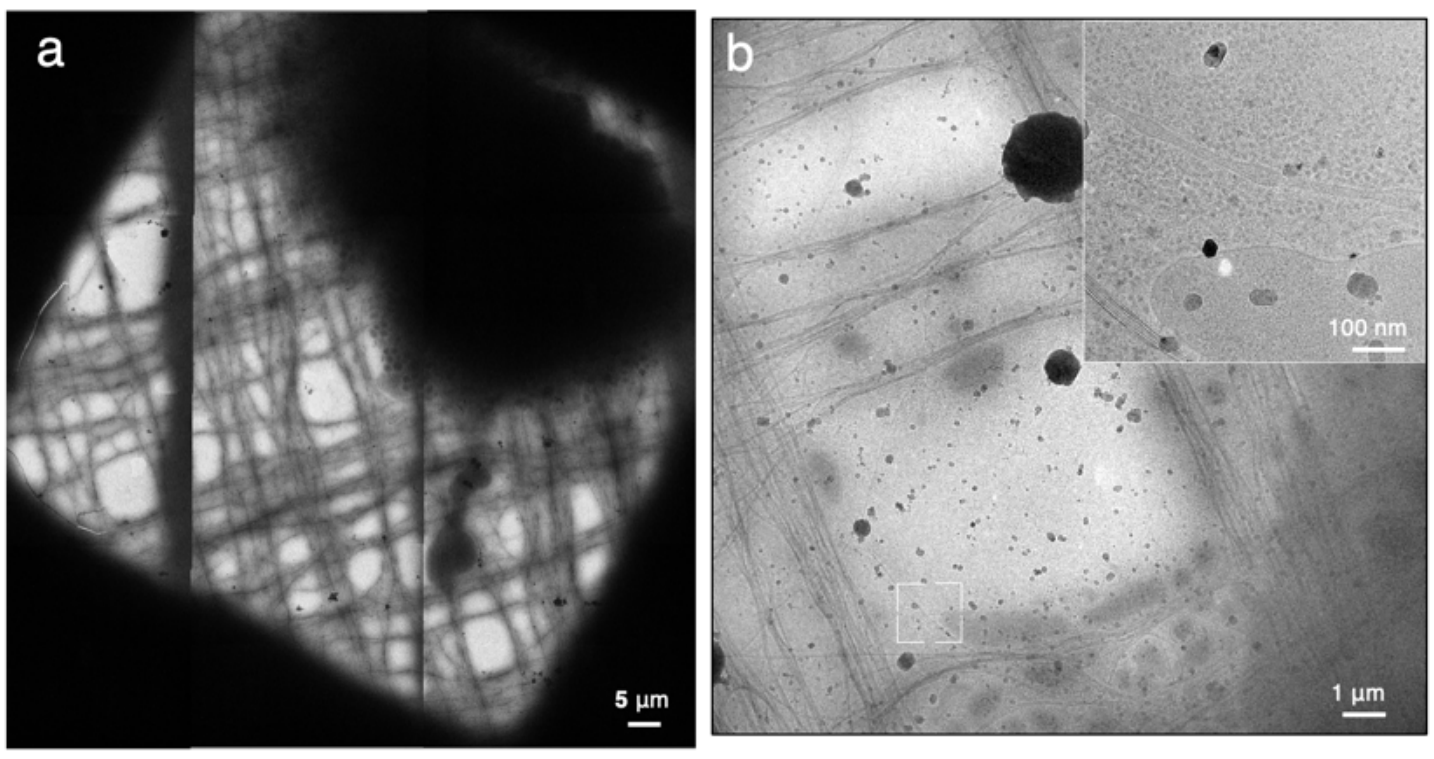

Supplementary Figure 16. Representative cryo-EM micrographs of NRK cells cultured on a CNT grid at different magnifications. More than 10 micrographs were captured independently with similar results.

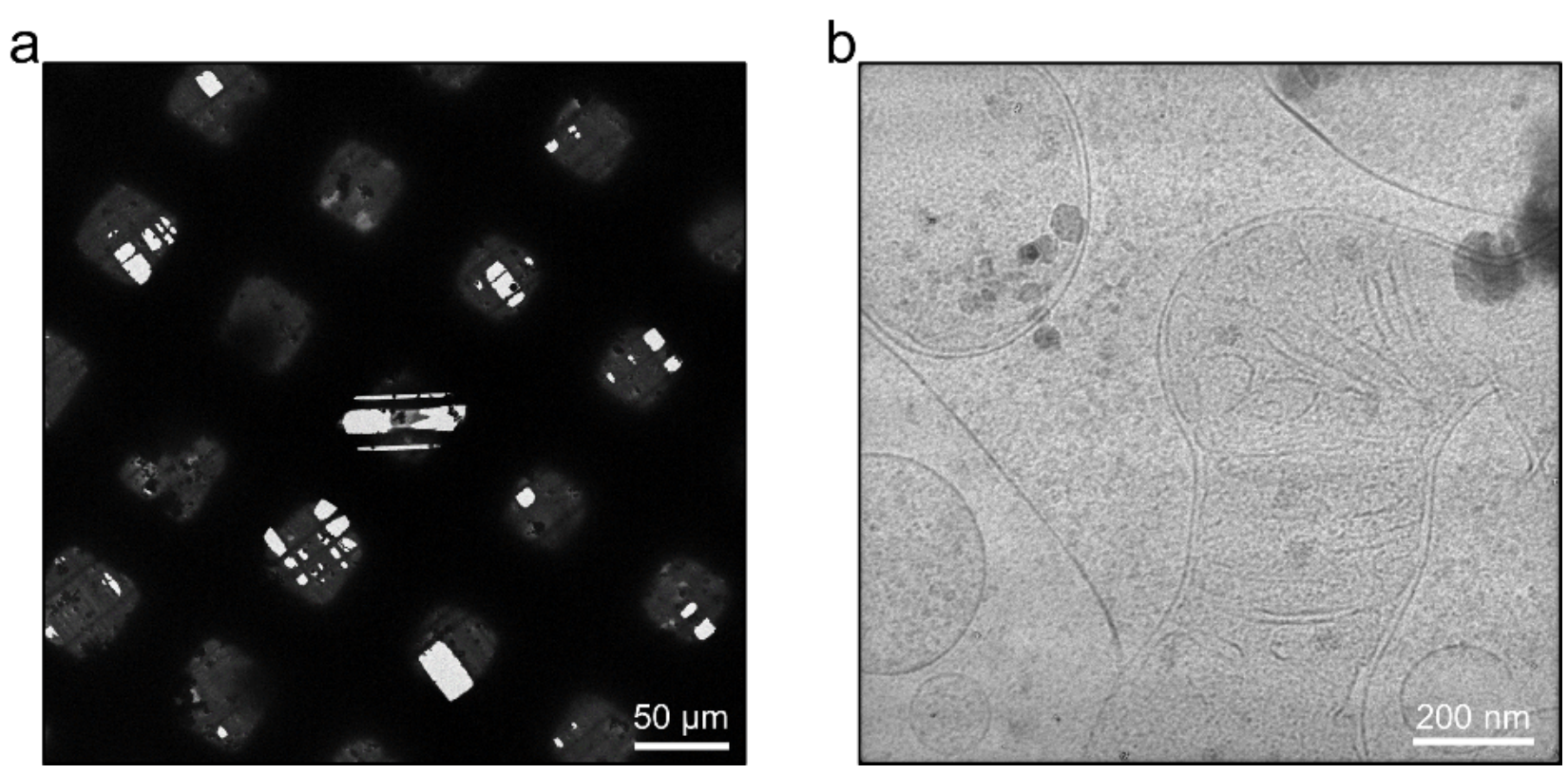

Supplementary Figure 17. Cryo-EM images of FIB-thinned lamellae from NRK cells on a CNT grid. a, Overview of a lamella. **b,** Higher-magnification view revealing distinct mitochondrial

structures. More than 3 grids were tested for each group independently with similar results.

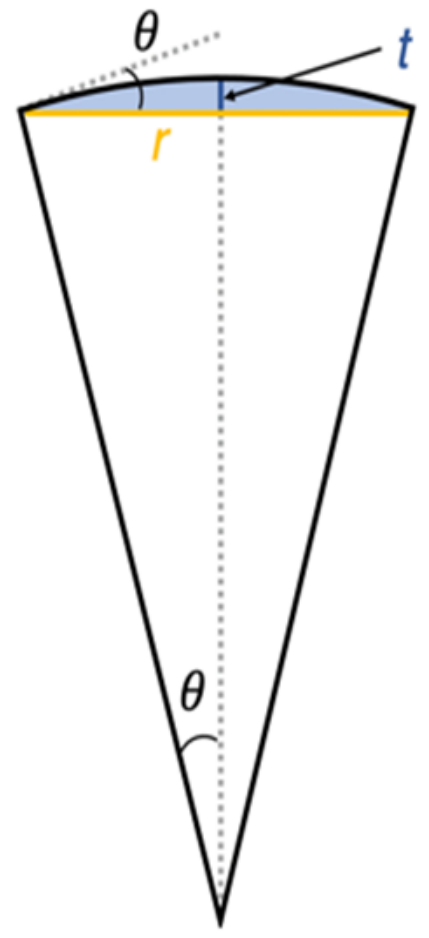

$$t = \frac{r}{sin\theta} - \frac{r}{tan\theta}$$

Supplementary Figure 18. The liquid thickness on a super-hydrophilic substrate surface. t, the thickness of the liquid film; *θ*, the water contact angle (5°); *r*, the radius of the substrate surface (50 μm). Based on the equation, the thickness (*t*) of the liquid film is calculated to be 2.18 μm.

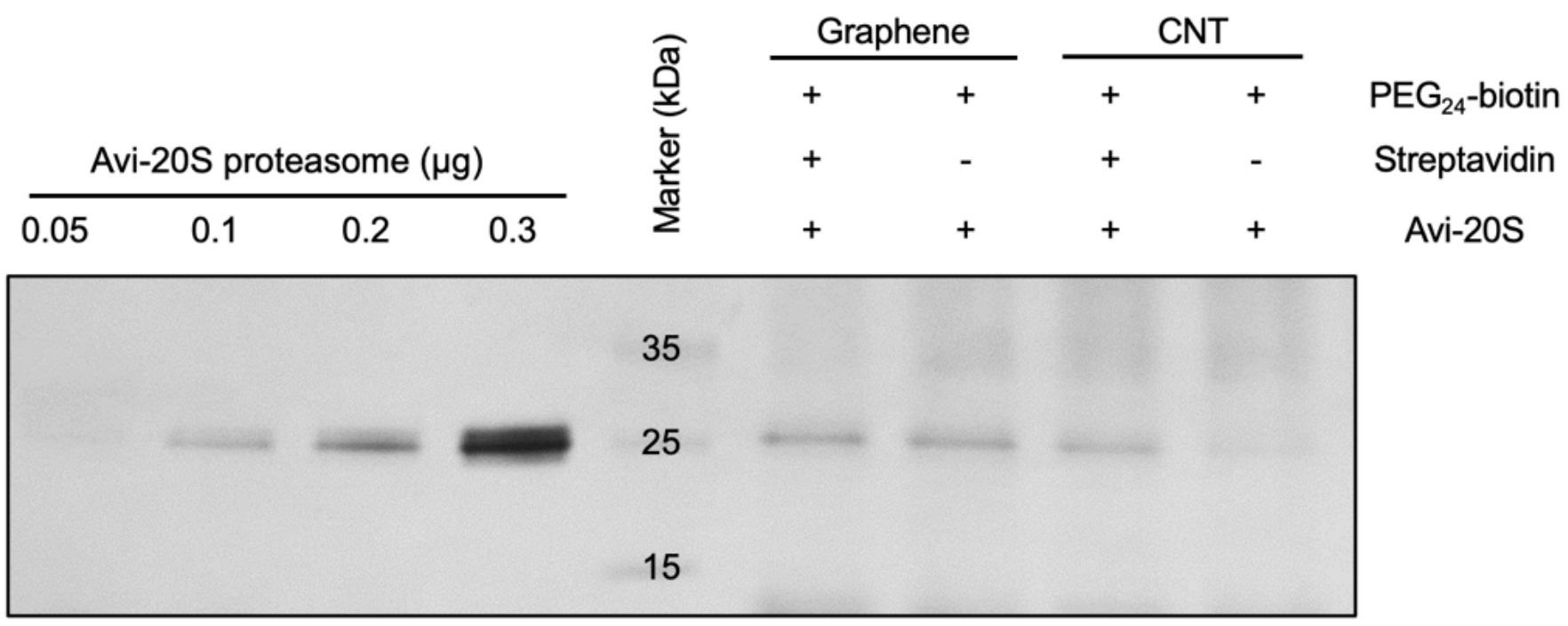

Supplementary Figure 19. Comparative analysis of binding specificity for CNT grids and graphene grids, using silver-stained SDS-PAGE gel. Lanes are loaded as follows (left to right): Avi-tagged 20S proteasome standards (0.05, 0.1, 0.2, 0.3 μg), protein marker, and samples from binding assays. For each grid type (PEG_{24}-biotin-modified graphene or CNT), two conditions were tested: incubation first with 0.3 μg streptavidin followed by 1 μg Avi-20S proteasome, and a control incubation with 0.3 μg Avi-20S proteasome alone. Three experiments were repeated independently with similar results.

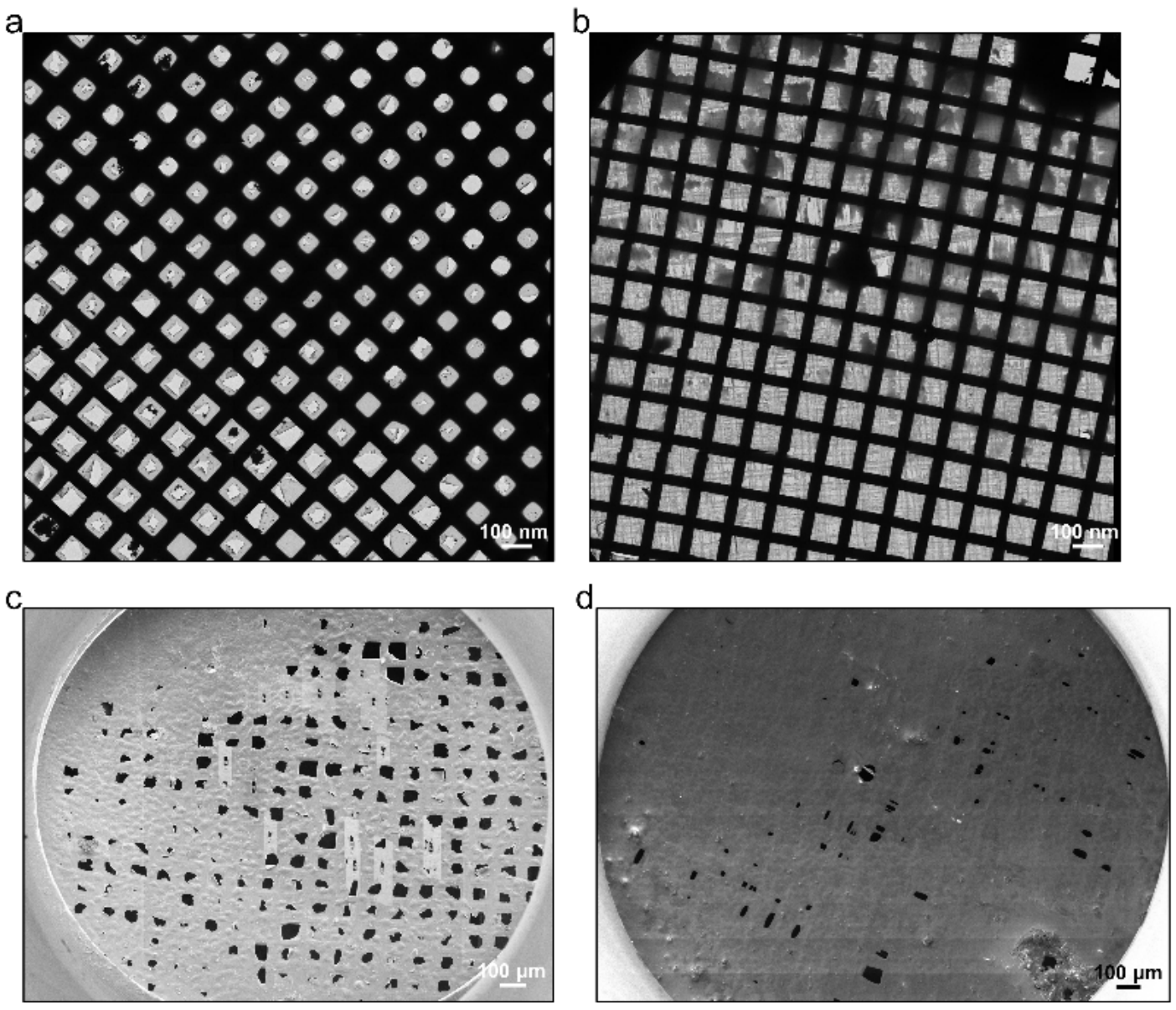

Supplementary Figure 20. Comparative imaging of conventional holey carbon-coated EM grids and CNT grids after vitrification. Cryo-EM (**a**) and SEM (**c**) images of a conventional holey carbon-coated EM grid. Cryo-EM (**b**) and SEM (**d**) images of a CNT grid. More than 3 grids were tested for each group independently with similar results. The SEM figure shown in 6c was cropped from (d) in this figure.

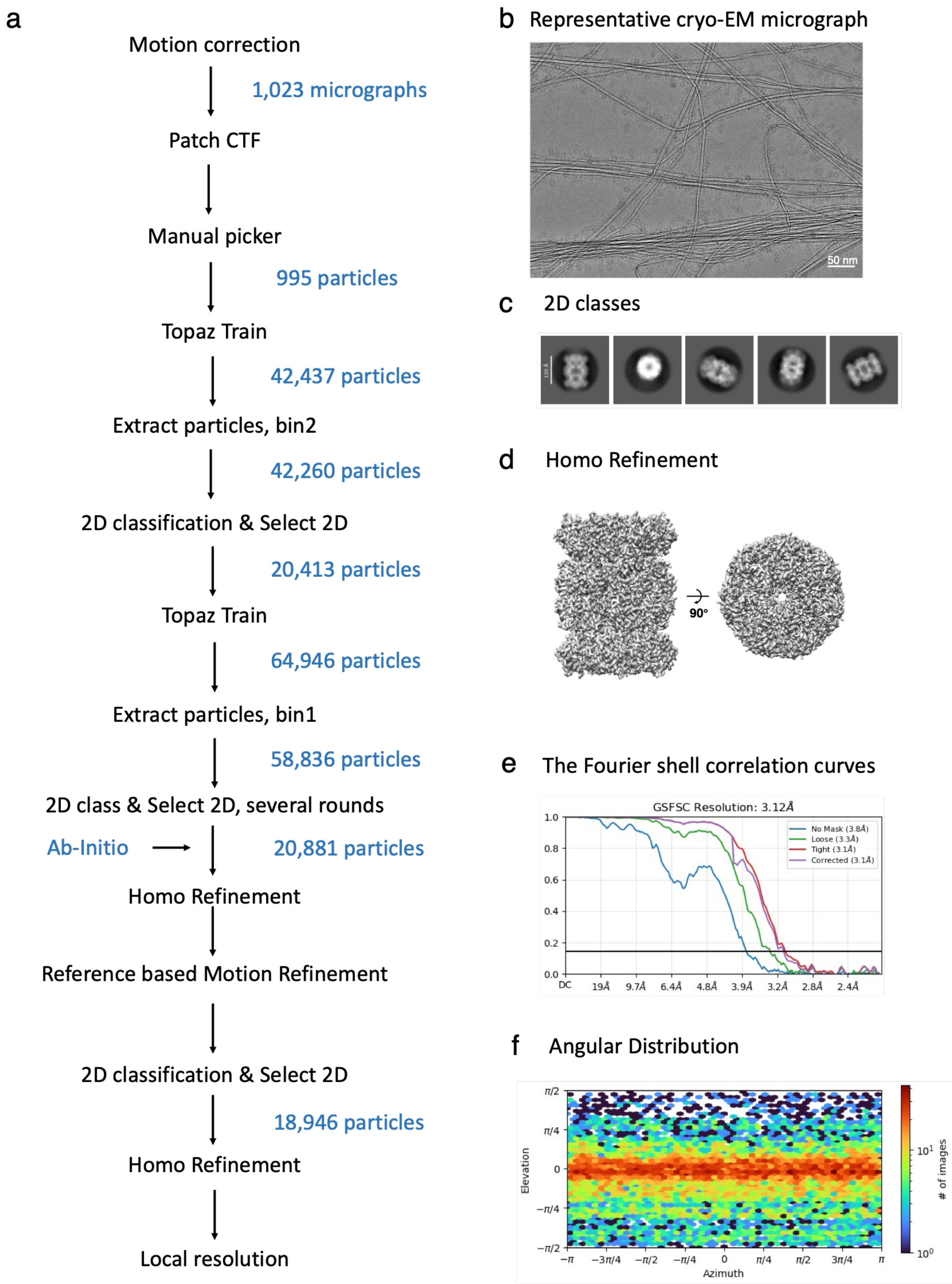

Supplementary Figure 21. Cryo-EM data-processing workflow of 20 proteasomes extracted from cell lysates using the PEG$_{24}$-biotin-modified CNT grid.

Supplementary Table 1. Cryo-EM data collection and reconstruction

	**GroES/GroEL on streptavidin-activated CNT**	**20S proteasome from cell lysates on streptavidin-activated CNT**	**GroES/GroEL from cell lysates on streptavidin-activated CNT**
**EMDB ID**	EMD-80845	EMD-80844	EMD-80843
**EMPAIR ID**	EMPIAR-13560	EMPIAR-13561	EMPIAR-13562
**Data collection and processing**			
Magnification	81,000	81,000	81,000
Voltage (kV)	300	300	300
Electron exposure (e^-/ Å^2)	50	50	50
Micrographs (no.)	1,032	1,023	1,149
Defocus range (µm)	0.7-2.4	0.7-2.5	0.6-2.4
Pixel size (Å)	1.0742	1.0742	1.0742
Symmetry imposed	C7	D7	C7
Final particle images (no.)	14,551	18,946	6,140
Map Resolution (Å)	3.4	3.1	4.1
FSC threshold	0.143	0.143	0.143

Source Data for Supplementary Figures (Uncropped image)

Supplementary Figure 11a

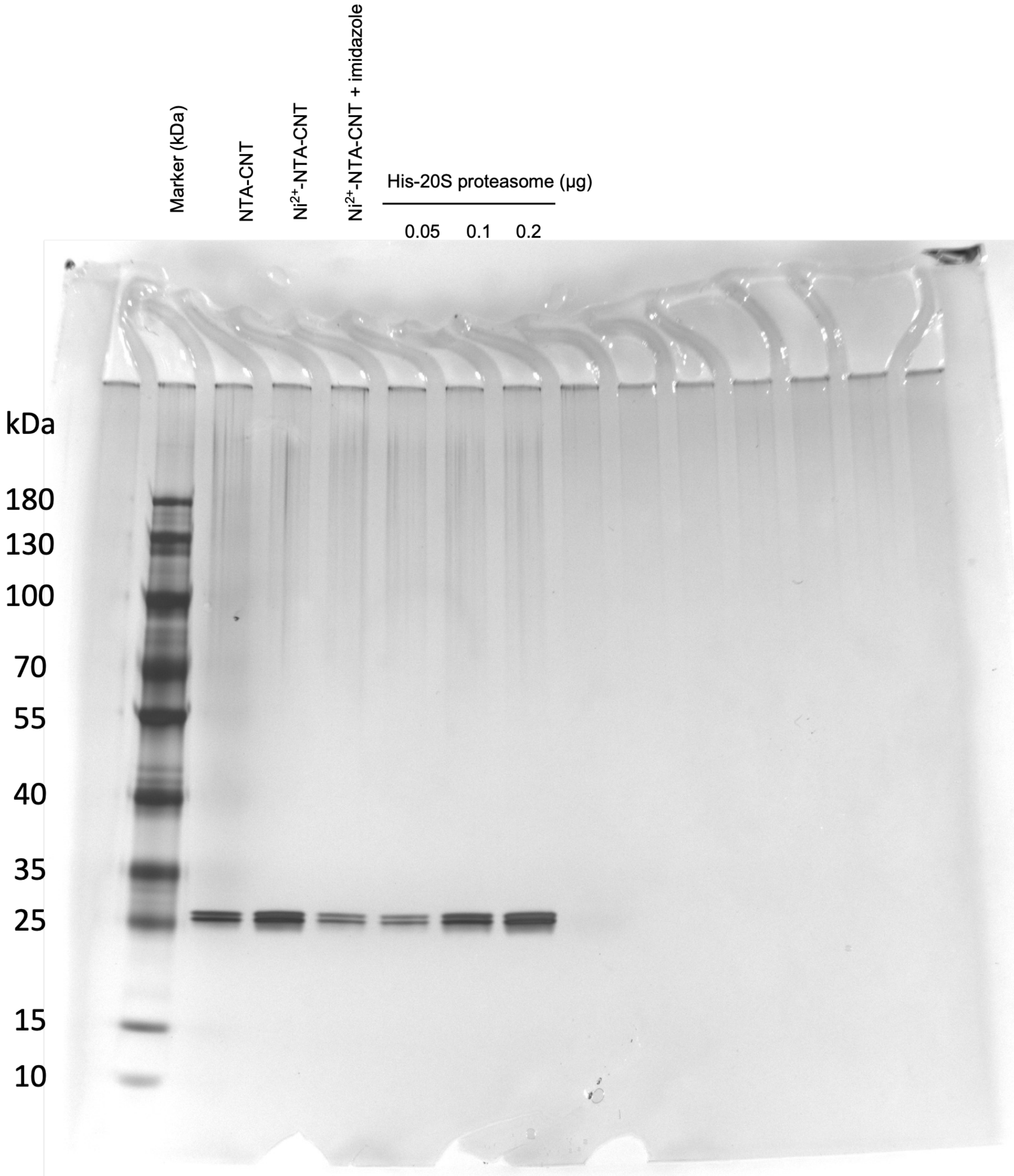

Supplementary Figure 19

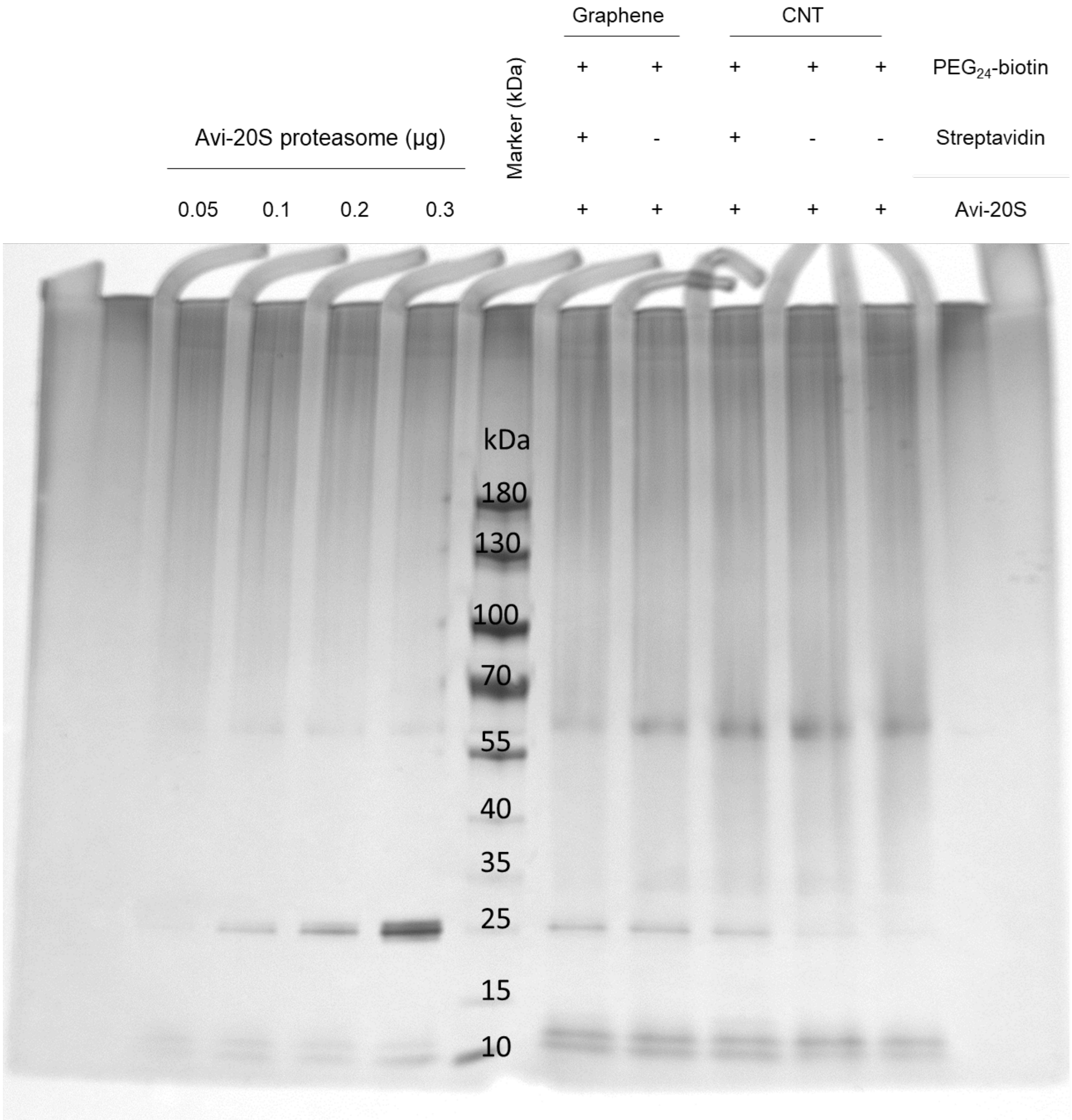

Supplement: Supplementary file 1 [file 41467_2026_77953_MOESM1_ESM.pdf]
