# Supplementary material for: Carbon nanotubes as the supporting film for cryo-EM specimen preparation
Source: Nat Commun. 2026 Sep 21;17:11016. doi: 10.1038/s41467-026-77953-8 (PMC13686963; doi:10.1038/s41467-026-77953-8)
Supplement: Supplementary file 2 [file 41467_2026_77953_MOESM2_ESM.pdf]

The Description of Additional Supplementary Files

Supplementary Video 1. Solution spreading on the CNT grid, implemented in VitroJet machine.

Supplementary Video 2. Solution spreading on the conventional holey carbon-coated grid, implemented in VitroJet machine.

Supplementary Video 3. The culture and migration of NRK cells on CNTs.

Supplementary Video 4. The culture and migration of NRK cells on glass slide.
