# Supplementary material for: Carbon nanotubes as the supporting film for cryo-EM specimen preparation
Source: Nat Commun. 2026 Sep 21;17:11016. doi: 10.1038/s41467-026-77953-8 (PMC13686963; doi:10.1038/s41467-026-77953-8)
Supplement: Supplementary file 3 [file 41467_2026_77953_MOESM7_ESM.pdf]

# nature portfolio

Corresponding author(s): Nan Liu
Last updated by author(s): Aug 9, 2026



# Reporting Summary

Nature Portfolio wishes to improve the reproducibility of the work that we publish. This form provides structure for consistency and transparency in reporting. For further information on Nature Portfolio policies, see our [Editorial Policies] and the [Editorial Policy Checklist].

## Statistics

For all statistical analyses, confirm that the following items are present in the figure legend, table legend, main text, or Methods section.

| n/a | Confirmed | |
|---|---|---|
| ☐ | ☒ | The exact sample size (*n*) for each experimental group/condition, given as a discrete number and unit of measurement |
| ☐ | ☒ | A statement on whether measurements were taken from distinct samples or whether the same sample was measured repeatedly |
| ☐ | ☒ | The statistical test(s) used AND whether they are one- or two-sided<br>*Only common tests should be described solely by name; describe more complex techniques in the Methods section.* |
| ☐ | ☒ | A description of all covariates tested |
| ☒ | ☐ | A description of any assumptions or corrections, such as tests of normality and adjustment for multiple comparisons |
| ☐ | ☒ | A full description of the statistical parameters including central tendency (e.g. means) or other basic estimates (e.g. regression coefficient) AND variation (e.g. standard deviation) or associated estimates of uncertainty (e.g. confidence intervals) |
| ☐ | ☒ | For null hypothesis testing, the test statistic (e.g. *F*, *t*, *r*) with confidence intervals, effect sizes, degrees of freedom and *P* value noted<br>*Give P values as exact values whenever suitable.* |
| ☒ | ☐ | For Bayesian analysis, information on the choice of priors and Markov chain Monte Carlo settings |
| ☒ | ☐ | For hierarchical and complex designs, identification of the appropriate level for tests and full reporting of outcomes |
| ☒ | ☐ | Estimates of effect sizes (e.g. Cohen's *d*, Pearson's *r*), indicating how they were calculated |

*Our web collection on statistics for biologists contains articles on many of the points above.*

## Software and code

Policy information about availability of computer code

| | |
|---|---|
| Data collection | We used EPU (version 3.2.0) software, SerialEM (version 3.8) software and AutoEMation software to collect single-particle cryo-EM dataset. The AutoEMation software was written by Dr. Jianlin Lei at Tsinghua University. We used SerialEM (version 3.8) software equipped with PACETomo (version 1.4.4) scripts to collect tilt series. |
| Data analysis | We used MotionCor2 (version 1.1.0) to correct the beam-induced motion of cryo-EM micrographs and used CryoSPARC (version 4.6.0) to perform 3D reconstruction. The CTF values of these motion-corrected micrographs were determined by CTFFIND4 algorithm (version 4.15) or PatchCTF in CryoSPARC (version 4.6.0). The structural analysis was performed in UCSF Chimera (version 1.16).<br>For cryo-ET reconstruction, we used Etomo (version 4.11) to align and reconstruct the tomograms. All these softwares are open-source. |

For manuscripts utilizing custom algorithms or software that are central to the research but not yet described in published literature, software must be made available to editors and reviewers. We strongly encourage code deposition in a community repository (e.g. GitHub). See the Nature Portfolio guidelines for submitting code & software for further information.

# Data

Policy information about availability of data

All manuscripts must include a data availability statement. This statement should provide the following information, where applicable:

- Accession codes, unique identifiers, or web links for publicly available datasets
- A description of any restrictions on data availability
- For clinical datasets or third party data, please ensure that the statement adheres to our policy

The cryo-EM density maps of GroES/GroEL on streptavidin-activated CNT, 20S proteasome from cell lysates on streptavidin-activated CNT and GroES/GroEL from cell lysates on streptavidin-activated CNT have been deposited into Electron Microscopy Data Bank (EMDB) under the accession numbers of EMD-80845 [https://www.ebi.ac.uk/pdbe/entry/emdb/EMD-80845], EMD-80844, [https://www.ebi.ac.uk/pdbe/entry/emdb/EMD-80844] and EMD-80843 [https://www.ebi.ac.uk/pdbe/entry/emdb/EMD-80843], respectively. The raw data of GroES/GroEL on streptavidin-activated CNT, 20S proteasome from cell lysates on streptavidin-activated CNT and GroES/GroEL from cell lysates on streptavidin-activated CNT have been deposited into the EMPIAR, with the corresponding accession codes EMPIAR-13560, EMPIAR-13561, EMPIAR-13562, respectively. Source data are provided with this paper. Source data are provided with this paper. The source data underlying Figures 2a, 3a, 3c-d, 3f, 5a-c, 5e-g and Supplementary Figures 2a-b, 4, 9a-b, and 10a-c are provided as a Source Data file.

## Research involving human participants, their data, or biological material

Policy information about studies with human participants or human data. See also policy information about sex, gender (identity/presentation), and sexual orientation and race, ethnicity and racism.

| | |
|---|---|
| Reporting on sex and gender | N/A |
| Reporting on race, ethnicity, or other socially relevant groupings | N/A |
| Population characteristics | N/A |
| Recruitment | N/A |
| Ethics oversight | N/A |

Note that full information on the approval of the study protocol must also be provided in the manuscript.

# Field-specific reporting

Please select the one below that is the best fit for your research. If you are not sure, read the appropriate sections before making your selection.

☒ Life sciences ☐ Behavioural & social sciences ☐ Ecological, evolutionary & environmental sciences

For a reference copy of the document with all sections, see nature.com/documents/nr-reporting-summary-flat.pdf

# Life sciences study design

All studies must disclose on these points even when the disclosure is negative.

| | |
|---|---|
| Sample size | For cryo-EM reconstruction, thousands of micrographs were recorded for each condition, with the exact numbers specified in the Methods section. To quantify particle density on CNTs, particles were counted across 200 nanotubes per condition. |
| Data exclusions | For cryo-EM reconstruction, particles grouped in bad classes with poorly defined features were excluded, because these particles were normally denatured or dissociated samples, which were harmful for high-resolution 3D reconstruction. |
| Replication | Water-contact angle measurements were repeated for 5 grids for each conditions (as indicated in Methods). All blots were repeated 3 times independently with similar results. Other replication were indicated in figure legends. |
| Randomization | Samples were allocated random, including the particle-motion and structural determination. Representative tilt-series were taken in a randomized way, we stochastically sampled the areas to record. |
| Blinding | For cryo-EM reconstruction in CryoSPARC (version 4.6.0), particles were randomly divided into two parts, and used for 3D structure determination. The consistence of structures generated by these two sub-datasets was used for the blinding test. The investigators were blinded to group allocation during data collection and/or analysis. |

# Reporting for specific materials, systems and methods

We require information from authors about some types of materials, experimental systems and methods used in many studies. Here, indicate whether each material, system or method listed is relevant to your study. If you are not sure if a list item applies to your research, read the appropriate section before selecting a response.

## Materials & experimental systems

| n/a | Involved in the study |
|---|---|
| ☒ | ☐ Antibodies |
| ☐ | ☒ Eukaryotic cell lines |
| ☒ | ☐ Palaeontology and archaeology |
| ☒ | ☐ Animals and other organisms |
| ☒ | ☐ Clinical data |
| ☒ | ☐ Dual use research of concern |
| ☒ | ☐ Plants |

## Methods

| n/a | Involved in the study |
|---|---|
| ☒ | ☐ ChIP-seq |
| ☒ | ☐ Flow cytometry |
| ☒ | ☐ MRI-based neuroimaging |

# Eukaryotic cell lines

Policy information about cell lines and Sex and Gender in Research

| | |
|---|---|
| Cell line source(s) | Rat NRK, gift from Laboratory of Li Yu, Tsinghua University. |
| Authentication | The cell lines were not authenticated. |
| Mycoplasma contamination | All the cell lines were routinely tested for mycoplasma and only when tested negative were used for the experiments. |
| Commonly misidentified lines (See ICLAC register) | No cell lines used in this study were found in the database of commonly misidentified cell lines that is maintained by ICLAC. |

# Plants

| | |
|---|---|
| Seed stocks | N/A |
| Novel plant genotypes | N/A |
| Authentication | N/A |
