# Supplementary material for: Carbon nanotubes as the supporting film for cryo-EM specimen preparation
Source: Nat Commun. 2026 Sep 21;17:11016. doi: 10.1038/s41467-026-77953-8 (PMC13686963; doi:10.1038/s41467-026-77953-8)
Supplement: Supplementary file 4 [file 41467_2026_77953_MOESM8_ESM.pdf]

nature portfolio

Peer Review File

Carbon nanotubes as the supporting film for cryo-EM specimen preparation

Corresponding Author: Dr Nan Liu

This file contains all reviewer reports in order by version, followed by all author rebuttals in order by version.

Version 0:

Reviewer comments:

Reviewer #1

(Remarks to the Author)
Review of NatureComms_26-013232-T

This is an interesting study of a novel technique for adding an additional support structure made of carbon nanotubes to a grid for cryoEM. It will be of interest to the field and may be useful for some specimens.

Major comments:

Particles bound to nanotubes will have a “missing wedge” with respect to the beam axis. When the particle is above or below the nanotube, it will no longer be useful to image since it will overlap. Please describe this in the paper.

Also, particles can be destroyed by the CNT- water interface just as they can be at the air-water interface. There seems to be sum evidence of this in the aggregation of some particles seen along the nanotubes in the micrographs. This should be discussed as well.

Minor points:

1. How did the presence of the CNT affect alignment of the particles (angular accuracy) in 2 & 3 D?

2. What was the Raman D/G ratio before and after plasma treatment?

3. Describe the CNT transfer procedure in detail with diagrams in the methods instead of just citing ref 27.

4. Particle in Fig 4C appear aggregated on surface of nanotube. Please characterise how many particles end up in the final reconstructions from this and other similar micrographs.

5. Suggest depositing dataset in EMPIAR.

6. Supplementary fig 1: please add a diagram of the copper frame with dimensions and a photo of the apparatus

7. Describe the laser and conditions used to cut in between the grids.

8. Supplementary fig 2: define the coordinates relative to the molecule in the plot, i.e. what is theta?

9. Cite Gusach et al. 2025 in paragraph 3 of introduction.

Reviewer #2

(Remarks to the Author)
The work by Jiling Song et al. describes the use of CNT films on TEM grids to improve liquid spreading and provide

additional nanoparticle orientations during TEM analysis. While the work is interesting, it appears largely routine when the structure of the present manuscript is compared with former publications by the same group on graphene-coated grids. The manuscript does compare Graphene and CNT-coated grids, but there are no real explanations about the possible differences in terms of selectivity, which may simply be due to differences in pre-treatment (UV-ozone, for example). The work demonstrating the compatibility between CNTs and cells, which makes direct cell culture on TEM grids possible, is also interesting. However, this has been known from the literature for quite some time.
Overall, even though the work is interesting and well conducted, I do not recommend publication in Nature Communications due to a lack of originality.

Version 1:

Reviewer comments:

Reviewer #1

(Remarks to the Author)
Well done on the revisions. No further comments.

Reviewer #2

(Remarks to the Author)
The reviewer aknowledges that the authors have made great efforts to revise the manuscript and answer the many questions raised by the reviewers. I can now align my decision with the other reviewer, even if I am not fully convinced that the current work is conceptually different enough to be completely original.

Open Access This Peer Review File is licensed under a Creative Commons Attribution 4.0 International License, which permits use, sharing, adaptation, distribution and reproduction in any medium or format, as long as you give appropriate credit to the original author(s) and the source, provide a link to the Creative Commons license, and indicate if changes were made.
In cases where reviewers are anonymous, credit should be given to 'Anonymous Referee' and the source.
The images or other third party material in this Peer Review File are included in the article's Creative Commons license, unless indicated otherwise in a credit line to the material. If material is not included in the article's Creative Commons license and your intended use is not permitted by statutory regulation or exceeds the permitted use, you will need to obtain permission directly from the copyright holder.
To view a copy of this license, visit https://creativecommons.org/licenses/by/4.0/

Reviewer #1 (Remarks to the Author):

Review of NatureComms_26-013232-T

This is an interesting study of a novel technique for adding an additional support structure made of carbon nanotubes to a grid for cryoEM. It will be of interest to the field and may be useful for some specimens.

R: We thank the reviewer for these positive comments and recognition of our work.

Major comments:

Particles bound to nanotubes will have a “missing wedge” with respect to the beam axis. When the particle is above or below the nanotube, it will no longer be useful to image since it will overlap. Please describe this in the paper.

R: We thank the reviewer for raising this point. Particles were automatically picked using Topaz and then selected through 2D and 3D classification to obtain the final set for structural reconstruction. Following the reviewer’s suggestion, we traced back the particles used for the final reconstruction and found that they are indeed distributed along the carbon nanotubes, rather than overlapping with them (**Figure R1**). We have now described this in our revised version as follows:

“*It’s worth noting that the particles used in the final reconstructions are distributed along the nanotubes and do not overlap with them.*” (Line 518)

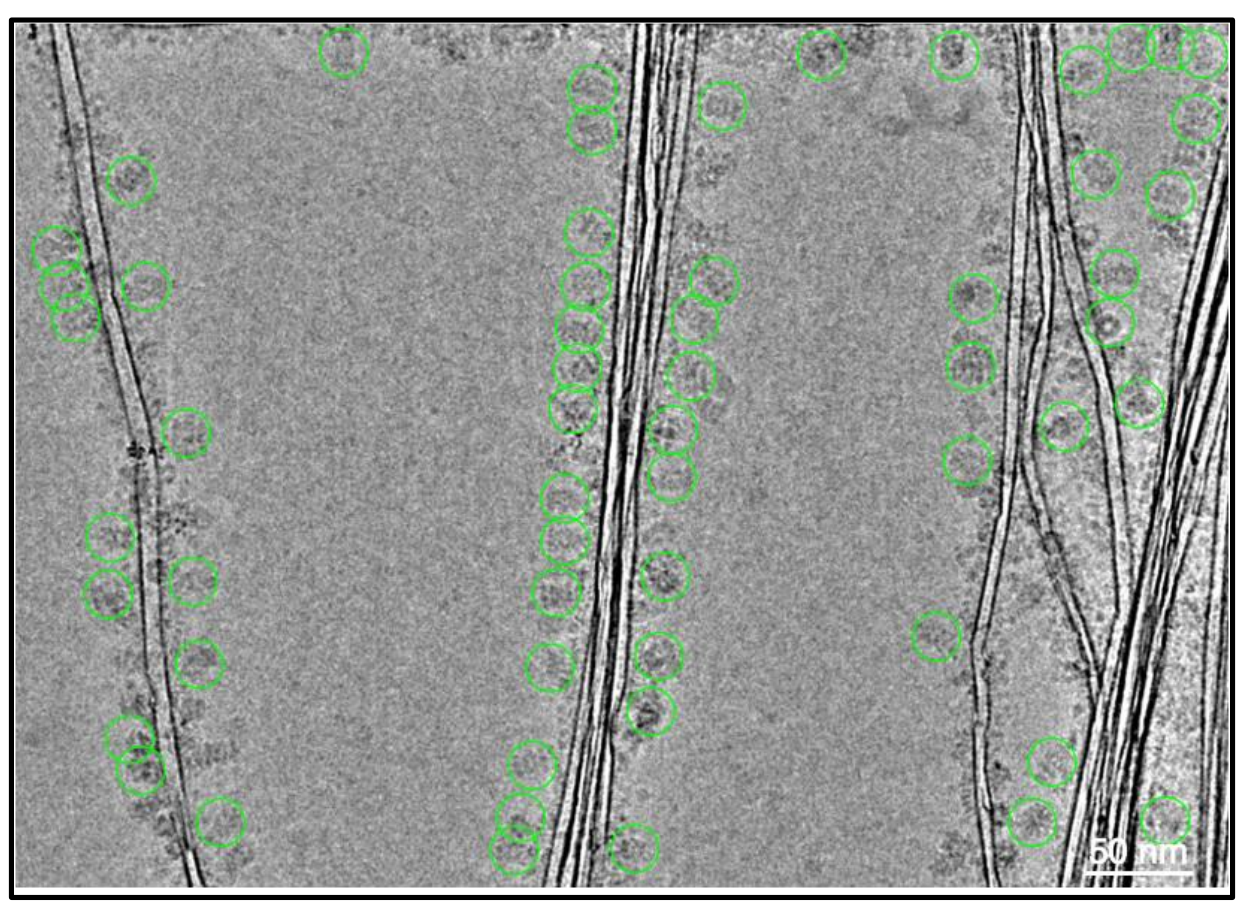

Figure R1: Representative micrograph showing particle positions (circled) that were used for the final structural reconstructions.

Also, particles can be destroyed by the CNT- water interface just as they can be at the air-water interface. There seems to be sum evidence of this in the aggregation of some particles seen along the nanotubes in the micrographs. This should be discussed as well.

R: We thank the reviewer for this important comment. We have added a sentence describing this to the revised manuscript, as below:

“*We also observed some protein aggregation along the carbon nanotubes (Figure 4c), which may result from suboptimal interactions at the CNT-water interface.*” (Line 202)

Minor points:

1. How did the presence of the CNT affect alignment of the particles (angular accuracy) in 2 & 3 D?

R: We thank the reviewer for raising this insightful question. During our data processing, we observed CNT signals appearing alongside the target protein particles in the 2D classification. We discarded particles in classes where the CNT clearly affected particle alignment and retained only those that displayed fine structural details. These high-quality particles were then used for 3D reconstruction, which showed no obvious effects from the CNT presence.

Following the reviewer’s suggestion, we tested several different parameters during 2D alignment in cryoSPARC to see if we could better eliminate CNT interference and optimize particle selection. Specifically, we focused on adjusting the mask diameter and whether to recenter particles (**Figure R2**). For 18,946 proteasome particles, using no mask with Re-center=*True* resulted in 40% (7,655 particles) being grouped in well-aligned classes, while the remainder exhibited strong CNT effects (**Figure R2a, top**). Using a larger-diameter mask (diameter of 210 Å) with Re-center=*True* increased the proportion of well-aligned particles to 55% (10,406 particles; **Figure R2a, middle**). The most effective results were obtained with a tight mask (diameter of 190 Å) and Re-center=*False*, which most effectively eliminated CNT interference during alignment. Under these conditions, 79% of particles (14,965) remained in high-quality classes (**Figure R2a, bottom**). Using these optimized parameters, we reprocessed our reconstructions, leading to significantly improved results (**Figure R2b–c**). For example, the reconstruction of GroEL/GroES on CNTs was refined to 3.4 Å resolution (**Figure R2c**), a substantial improvement over our previous reconstruction at 4.3 Å resolution. These updates have been incorporated into the revised Figure 4 and Figure 5. And the alignment parameters are also added into the revised Methods, as described below:

“*In 2D classification, we disabled the “Re-center” option and applied a tight mask with a diameter slightly larger than that of the target particle. We found that this approach best eliminated CNT interference during alignment and yielded more usable particles for subsequent reconstruction*.” (Line 510)

Once again, we thank the reviewer for this insightful question that greatly improved our manuscript.

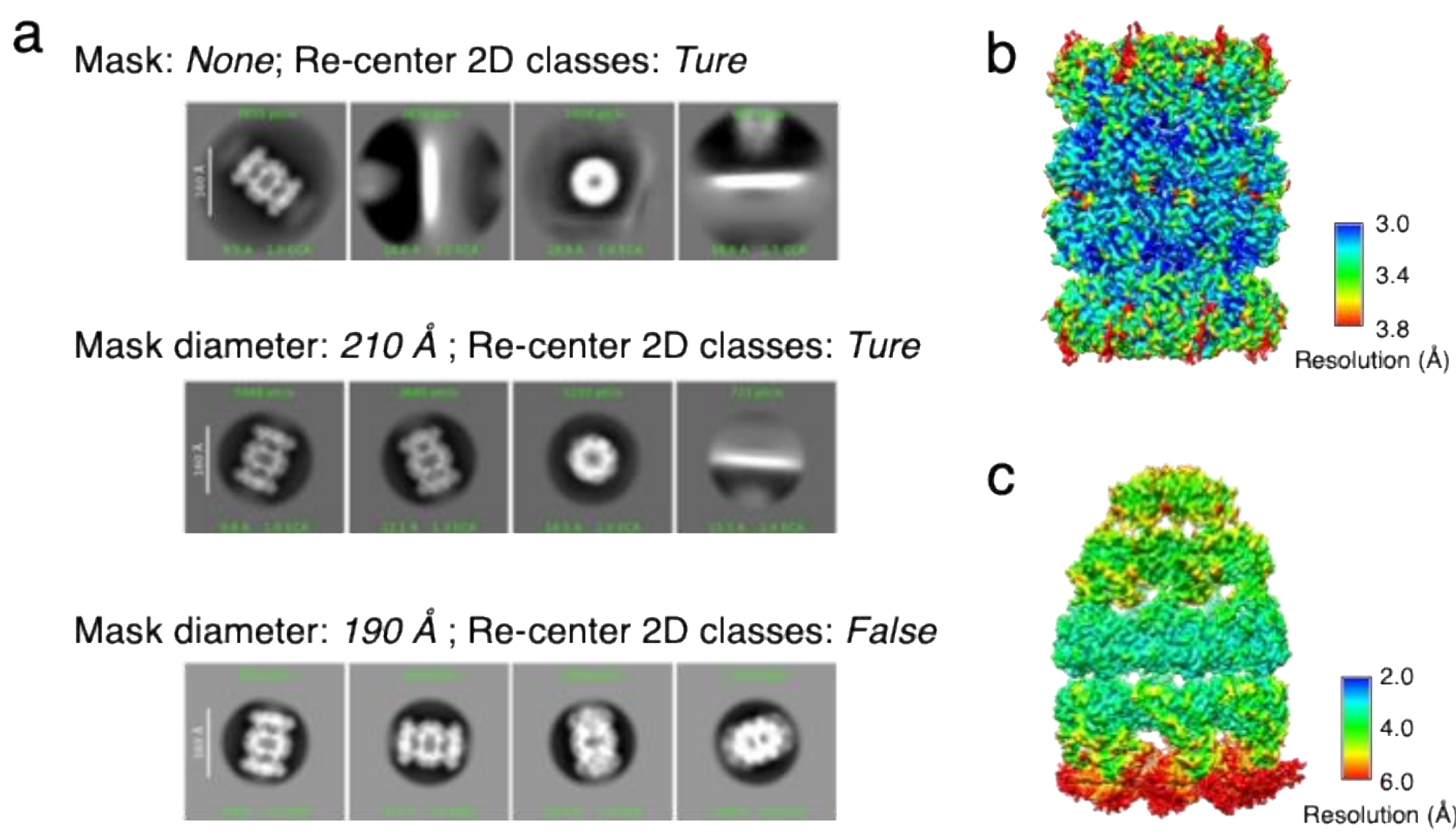

Figure R2: Optimization of alignment parameters. (**a**) Testing of mask and "Re-center" options for 20S proteasome particle alignment. (**b–c**) Using a tight mask and disabling the "Re-center" option in 2D classification led to better particle selection and improved reconstructions for the 20S proteasome (**b**) and the GroES/GroEL complex (**c**).

2. What was the Raman D/G ratio before and after plasma treatment?

R: We apologize for not clearly indicating the Raman D/G intensity ratios in Supplementary Figure 10. These ratios are 0.53 before plasma treatment, 0.83 for Ni^{2+}-NTA-modified CNTs, and 0.91 for biotin-modified CNTs (**Figure R3a**), which align well with documented Raman D/G ratios for both pristine and functionalized CNTs (1. W. S. Kim et al., 2007, doi: 10.1109/IVNC.2007.4480960; 2. S. Lee et al., 2008, doi: 10.1016/j.carbon.2008.09.029). Additionally, we also characterized plasma-treated CNTs at both O_2 and Ar atmospheres and obtained the averaged D/G intensity ratios of 0.72 and 0.81 (**Figure R3a-b**).

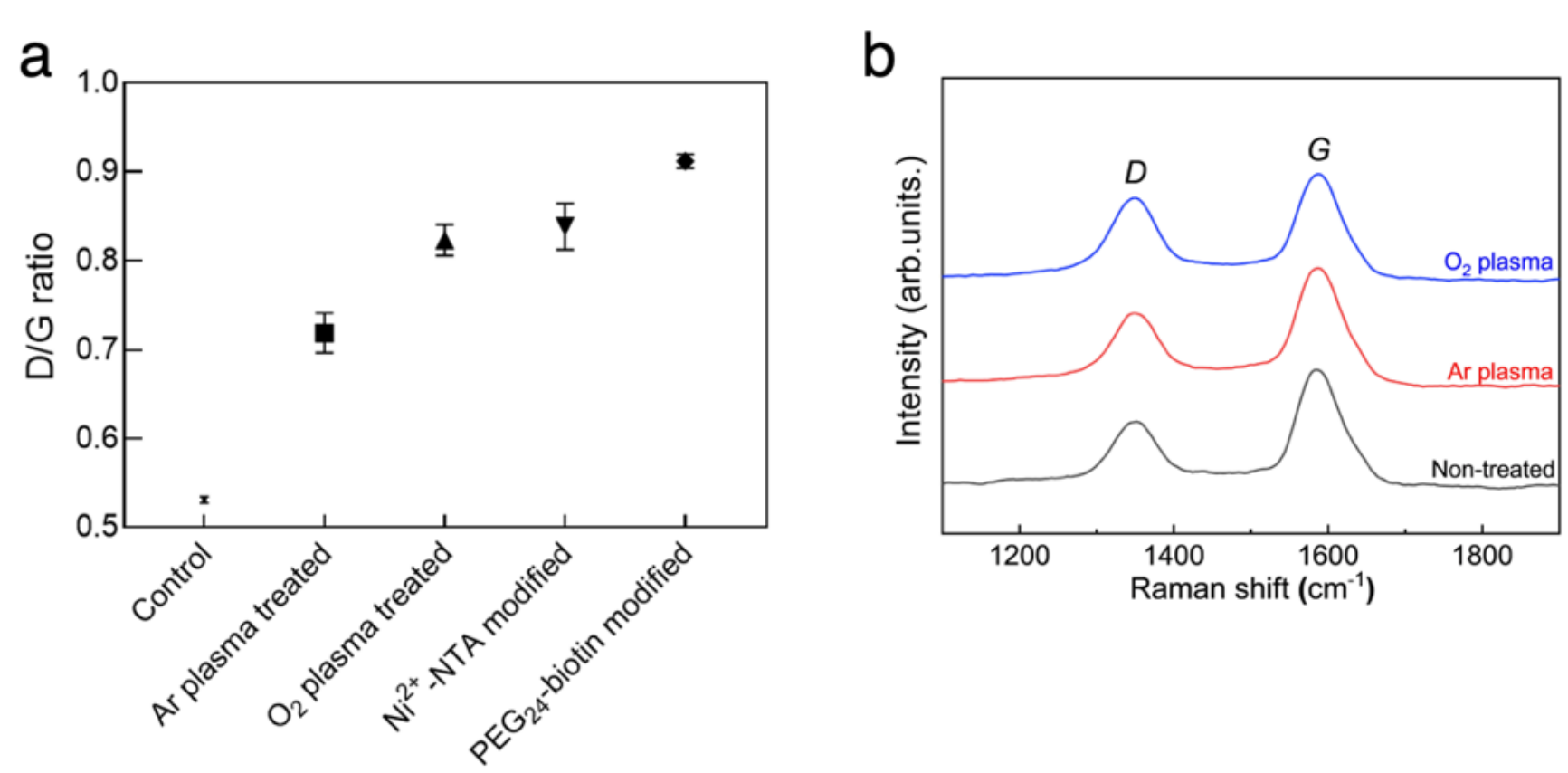

Figure R3: Raman characterization of CNTs. (**a, Supplementary Figure 10c in revised manuscript**) Average Raman D/G intensity ratios for non-treated CNTs (Control), plasma-treated

CNTs under Ar atmosphere and O_2 atmosphere, Ni^{2+}-NTA-modified CNTs, and PEG_{24}-biotin-modified CNTs. The averaged ratios are 0.53, 0.72, 0.81, 0.83, and 0.91, respectively. For each condition, measurements were repeated 3 times. (**b, Supplementary Figure 10b**) Representative Raman spectra of CNTs with and without plasma treatment.

The above information has now been included in our revised manuscript, as the revised Supplementary Figure 10b-c (legends shown below). The original values used for plotting the Raman curves have been provided in the Source Data file.

"***Supplementary Figure 10. Raman characterization of CNTs. a,*** *Representative Raman spectra of non-treated CNTs (black), PEG_{24}-biotin-modified CNTs (red), and Ni^{2+}-NTA-modified CNTs (blue).* ***b,*** *Representative Raman spectra of non-treated CNTs (black), plasma-treated CNTs under Ar atmosphere (red) and O_2 atmosphere (blue).* ***c****, Average Raman D/G intensity ratios for non-treated CNTs (Control), plasma-treated CNTs under Ar atmosphere and O_2 atmosphere, Ni^{2+}-NTA-modified CNTs, and PEG_{24}-biotin-modified CNTs. The ratios are 0.53, 0.72, 0.81, 0.83, and 0.91, respectively. For each condition, measurements were repeated 3 times.*"

3. Describe the CNT transfer procedure in detail with diagrams in the methods instead of just citing ref 27.

R: We thank the reviewer for this suggestion. We have revised the CNT transfer procedure diagram by including additional details and photographs of the actual apparatus alongside the schematic diagrams (**Figure R4**). Furthermore, we have revised the description of the CNT transfer procedure in the Methods section, as detailed below:

"*A single-layer carbon nanotube (CNT) film was obtained from the SACNT arrays using the established procedure[28]. Specifically, a single layer of CNT film was drawn from the SACNT array using a plastic rod, and a second layer was drawn on top of it. These two layers of CNT films were then arranged in a vertically crossed pattern on a flat glass slide substrate (Supplementary Figure 1). This substrate was later immersed into a container filled with deionized water, allowing the crossed CNT films to float on the water's surface….*" (Line 315)

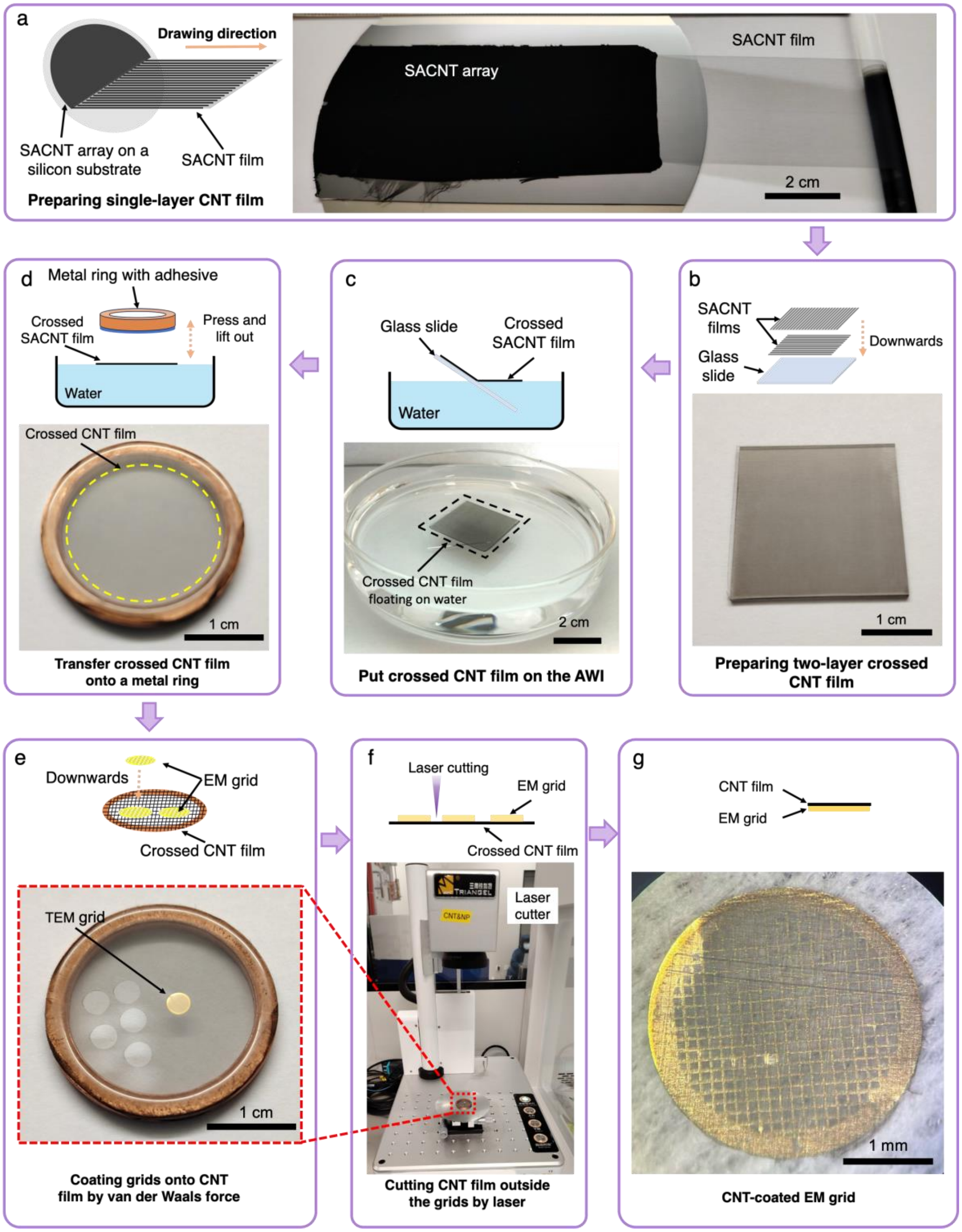

Figure R4 (revised Supplementary Figure 1). Schematic of the preparation procedure of CNT grids. A single-layer CNT film was drawn from the SACNT arrays (**a**), followed by arranging a second layer of the CNT film onto the first CNT film in a vertically crossed pattern (**b**). The crossed CNT films were then floated on the water's surface (**c**), with a copper ring used to lift them out of the water (**d**). Subsequently, EM grids were placed onto the crossed CNT films (**e**),

and a laser cutter was utilized to trim the excess CNTs outside the EM grids (**f**) to obtain the CNT-coated EM grids (**g**).

4. Particle in Fig 4C appear aggregated on surface of nanotube. Please characterise how many particles end up in the final reconstructions from this and other similar micrographs.

R: We thank the reviewer for this suggestion. For the GroEL/GroES reconstruction using streptavidin-activated CNTs, we initially used Topaz to pick 56,508 particles from the micrographs, and 14,551 (~ 26%) were used for the final reconstruction. For the GroEL/GroES reconstruction using PEG_{24}-biotin-modified CNTs, we initially used Topaz to pick 80,347 particles from the open region of micrographs, and 19,064 (~24%) were used for the final reconstruction.

5. Suggest depositing dataset in EMPIAR.

R: We thank the reviewer for this suggestion. We have deposited the raw data of GroEL/GroES on streptavidin-activated CNT, 20S proteasome from cell lysates on streptavidin-activated CNT and GroEL-GroES from cell lysates on streptavidin-activated CNT into EMPIAR, under the session numbers EMPIAR-13560, EMPIAR-13561, EMPIAR-13562, respectively. This information has been updated in the revised Data availability section (Line 548).

6. Supplementary fig 1: please add a diagram of the copper frame with dimensions and a photo of the apparatus

R: We thank the reviewer for this suggestion. We have added these photographs with scale bars alongside the schematic diagrams for each step in the revised Supplementary Figure 1, as below.

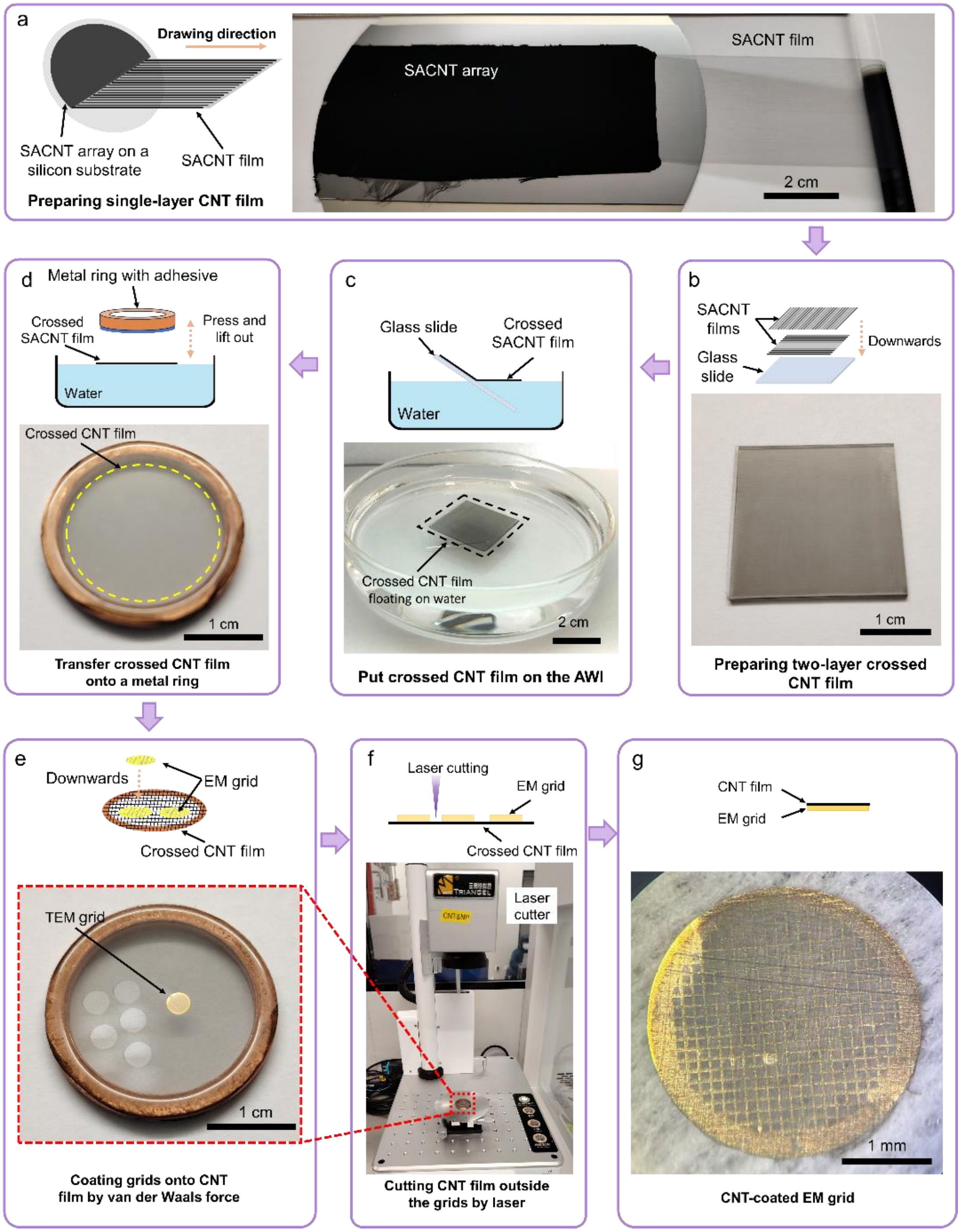

Figure R4 (revised Supplementary Figure 1). Schematic of the preparation procedure of CNT grids. A single-layer CNT film was drawn from the SACNT arrays (**a**), followed by arranging a second layer of the CNT film onto the first CNT film in a vertically crossed pattern (**b**). The crossed CNT films were then floated on the water's surface (**c**), with a copper ring used to lift them out of the water (**d**). Subsequently, EM grids were placed onto the crossed CNT films (**e**), and a laser cutter was utilized to trim the excess CNTs outside the EM grids (**f**) to obtain the CNT-coated EM grids (**g**).

7. Describe the laser and conditions used to cut in between the grids.

R: We thank the reviewer for this suggestion. We employed a commercial YAG fiber laser cutting equipment to process carbon nanotube (CNT) films, with a laser wavelength of 1.06 µm, a laser power of approximately 20 W, and a cutting speed of 1.5 m/s. We have incorporated these in our revised Method section.

"*A commercial YAG fiber laser cutting equipment, with a wavelength of 1.06 µm, was used to cut the CNT films at a laser speed of 1.5 m/s and with the laser power set to approximately 20 W (as shown in Supplementary Figure 1).*" (Line 326)

8. Supplementary fig 2: define the coordinates relative to the molecule in the plot, i.e. what is theta?

R: We thank the reviewer for pointing this out. We believe the reviewer may be referring to Figure 2d, which illustrates particle orientation using the Euler angles theta (θ) and phi (φ). In contrast, Supplementary Figure 2 shows the mechanical properties of CNTs.

We apologize for not clearly describing the coordinates in the orientation distribution figure. Theta (θ) and phi (φ) are Euler angles that define the view direction of each particle, assigned during 3D alignment relative to the reference model. Additionally, the size of each spot in the plot represents the relative population of particles corresponding to that particular view. To make this clearer, we have added the following sentence to the Fig. 2d caption:

"*In the particle orientation distribution plot, the Euler angles θ and Φ define the orientation of each particle relative to the reference model during 3D alignment; larger spot sizes colored in red indicate more particles assigned to that view*." (Line 740)

9. Cite Gusach et al. 2025 in paragraph 3 of introduction.

R: We thank the reviewer for bringing this insightful work to our attention. We have added it in the introduction, as below:

"*A recent approach involves spraying a small sample droplet at high speed onto EM grids that are pre-cooled to liquid ethane temperature, allowing the sample to be thinned and vitrified within microseconds[22]. This method eliminates the exposure of protein particles to the air–water interface*." (Line 59)

22 Gusach, A., Sader, K. & Russo, C. J. Outrunning protein diffusion to the air-water interface in cryoEM. *Proc Natl Acad Sci U S A* **122**, e2516900122 (2025). https://doi.org:10.1073/pnas.2516900122

Reviewer #2 (Remarks to the Author):

The work by Jiling Song et al. describes the use of CNT films on TEM grids to improve liquid spreading and provide additional nanoparticle orientations during TEM analysis. While the work is interesting, it appears largely routine when the structure of the present manuscript is compared with former publications by the same group on graphene-coated grids. The manuscript does compare Graphene and CNT-coated grids, but there are no real explanations about the possible differences in terms of selectivity, which may simply be due to differences in pre-treatment (UV-ozone, for example). The work demonstrating the compatibility between CNTs and cells, which makes direct cell culture on TEM grids possible, is also interesting. However, this has been known from the literature for quite some time.

Overall, even though the work is interesting and well conducted, I do not recommend publication in Nature Communications due to a lack of originality.

R: We sincerely thank the reviewer for recognizing our work as interesting and well-conducted, and for providing these constructive comments.

We understand the reviewer's concern regarding originality when comparing this work to our previous publications on graphene-coated grids. However, we would like to clarify that the design principle and physical behavior of 1D carbon nanotubes (CNTs) are fundamentally distinct from 2D graphene, leading to several unique advantages in cryo-EM, as detailed below.

First, unlike graphene, the 1D CNT network exerts unique capillary forces that improve solution spreading on the grids. This property is highly advantageous and uniquely positions CNT grids for use with emerging spray-based specimen preparation methods. Second, in our previous work with 2D graphene, we often encountered preferential orientation issue, which required us to design varied surface modifications to alleviate it (Lu, Y. et al., 2022, doi: 10.1038/s41467-022-34579-w; Cheng, H. et al., 2023, doi: 10.1021/jacs.3c00659). In contrast, CNTs inherently resolve preferential orientation problems far more effectively.

Regarding the reviewer's question on selectivity, we apologize for the lack of clarity in our initial description of the modification procedures. Both the graphene and CNTs used in our experiments (Supplementary Figure 19) underwent identical modification procedures. Specifically, both were first glow-discharged under an argon atmosphere and subsequently incubated with a diazonium PEG_{24}-biotin solution.

To further demonstrate the differences in binding specificity between functionalized CNTs and graphene, and in response to the reviewer's helpful suggestion, we tested another commonly used functionalization method (NTA-Ni^{2+}). Following previously established protocol (Cheng H, et al., 2023, doi: 10.1021/jacs.3c00659), both CNT and graphene grids were glow-discharged in an oxygen atmosphere and functionalized with PEG_{44}-NTA-Ni^{2+}. We then applied His-tagged 20S proteasome to the PEG_{44}-NTA-Ni^{2+} functionalized CNT and graphene grids, using PEG_{44}-NTA functionalized grids (without Ni^{2+}) as negative controls. The results were highly consistent (**Figure R5**): in the absence of Ni^{2+}, the CNTs exhibited minimal binding of His-tagged 20S proteasomes,

whereas graphene showed noticeable background binding. In the presence of Ni^{2+}, both the CNTs and graphene demonstrated a high binding capacity for the target proteins, with the CNTs showing higher binding levels.

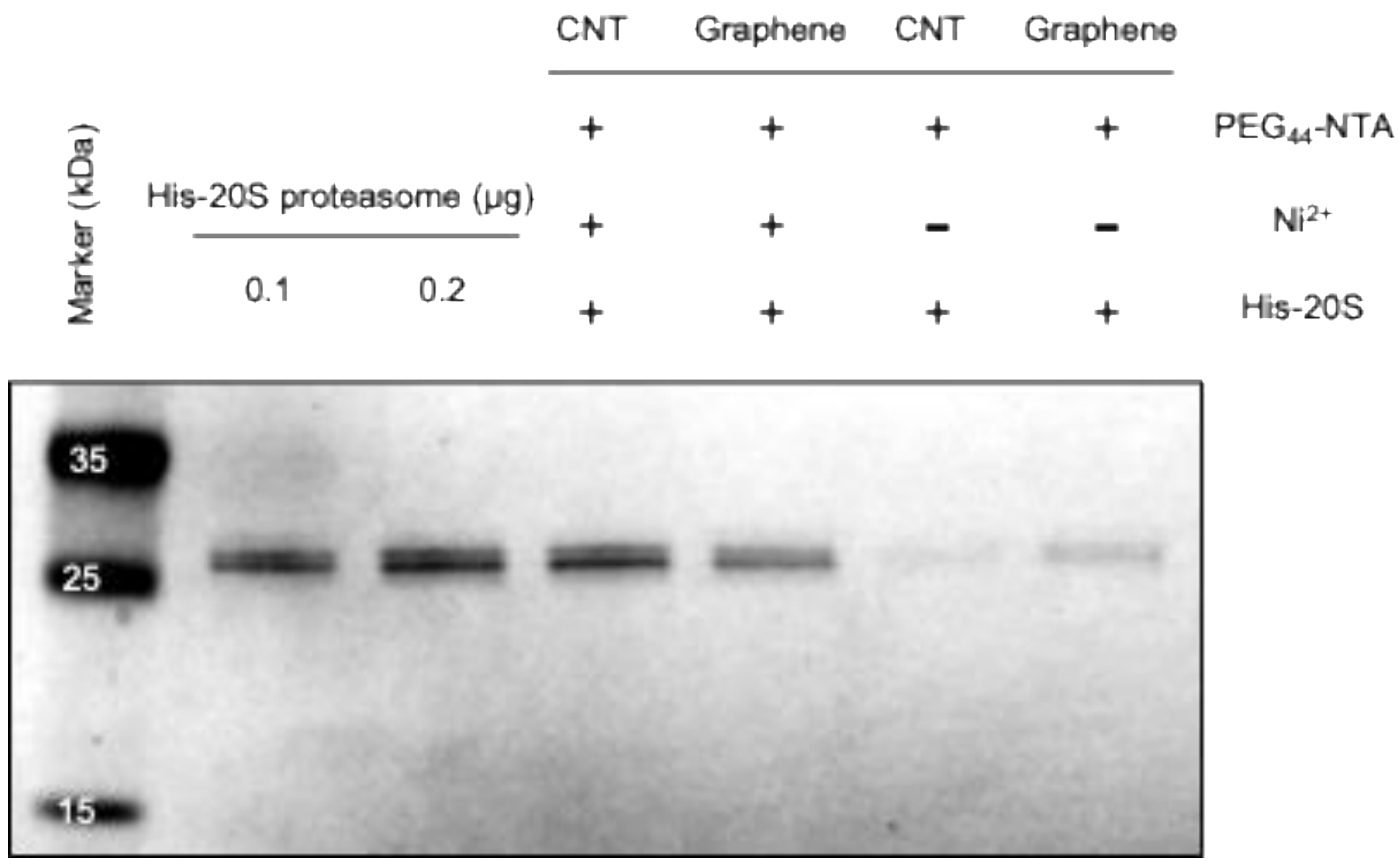

Figure R5. The binding specificity of PEG_{44}-NTA-Ni^{2+} functionalized CNT and graphene grids, prepared via the same functionalization procedure, was compared using silver-stained SDS-PAGE.

We attribute the observed differences in selectivity to the distinct properties of the two materials, which has been discussed in previous studies on protein adsorption onto carbon nanomaterials (such as: 1. Sengupta et al., 2016, doi: 10.1039/C5RA15007H; 2. Marchesa and Prato, 2015, doi: 10.1039/C4CC09173F; 3. Mu et al., 2014, doi: 10.1021/cr400295a). Physicochemical interactions with nanomaterials can induce protein unfolding and conformational changes, driving the tight binding of proteins onto the nanomaterial surface (1. Sengupta et al., 2016, doi: 10.1039/C5RA15007H; 2. Nel, A. et al., 2009, doi: 10.1038/nmat2442). The planar surface of graphene maximizes the available contact area with proteins, promoting their unfolding and flattening onto the surface, which makes subsequent washing steps difficult. In contrast, the highly curved, cylindrical surface of CNTs—especially those with small diameters—restricts the protein–surface contact area. This restricts stable non-specific adsorption, ensuring proteins are only retained if specifically captured by the modifiers on the CNTs. Consequently, the CNT platform exhibits better binding specificity, enabling the isolation of target proteins from cell lysates for cryo-EM structural analysis—an outcome we could not achieve with graphene.

We have added a sentence detailing the graphene treatment in the revised Method section, and incorporated the above discussion in the revised Discussion section, as shown below:

"…, which was then subjected to a glow discharge for 10 s under an argon gas flow of 5 sccm in a plasma cleaning system (Gatan Solarus),…" (Line 377)

"*We attribute the selectivity differences to the intrinsic properties of the two materials. The planar structure of graphene maximizes the contact area and binding strength with all input proteins, promoting non-specific adsorption. In contrast, the highly curved, cylindrical surface of CNTs—especially those with small diameters—restricts the protein–surface contact area, thereby preventing stable adsorption unless the target proteins are specifically captured by the modifiers on the CNTs*." (Line 292)

Finally, we agree that the physicochemical characterization of CNTs and their application in cell culture have been documented previously (such as: 1. Wei W, et al., 2007, doi: 10.1166/jnn.2007.655; 2. Ponti J, et al., 2010, doi: 10.1016/j.toxlet.2010.04.018; 3. Suarato G, et al., 2024, doi: 10.1021/acsami.3c14527.). However, we respectfully hope the reviewer will appreciate the primary novelty of our work. For the first time, we have systematically explored the use of CNTs and their diverse bioactive modifications as a comprehensive cryo-EM platform. In this study, we have demonstrated that the unique capillary action of CNTs facilitates solution thinning during cryo-EM specimen preparation, characterized how CNTs and their modifications affect protein interactions and/or purification for structural reconstruction, and shown their integration with cellular structural investigation. We would deeply appreciate it if the reviewer could acknowledge these innovations and recognize that our comprehensive characterization of CNTs as supports for biological cryo-EM structural studies represents a high degree of novelty.

Reviewer #1 (Remarks to the Author):

Well done on the revisions. No further comments.

R: We thank the reviewer for the encouraging and positive assessment of our revisions. We greatly appreciate the reviewer's time and expertise in evaluating our work.

Reviewer #2 (Remarks to the Author):

The reviewer acknowledges that the authors have made great efforts to revise the manuscript and answer the many questions raised by the reviewers. I can now align my decision with the other reviewer, even if I am not fully convinced that the current work is conceptually different enough to be completely original.

R: We thank the reviewer for acknowledging the substantial efforts we have made in revising the manuscript. We are pleased that the reviewer is now willing to align with the favorable decision of the other reviewer, and we greatly appreciate the reviewer’s constructive and thoughtful engagement.
